# Tumor-secreted IFI35 promotes proliferation and cytotoxic activity of CD8^+^ T cells through PI3K/AKT/mTOR signaling pathway in colorectal cancer

**DOI:** 10.1186/s12929-023-00930-6

**Published:** 2023-06-28

**Authors:** Peisi Li, Dawang Zhou, Dongwen Chen, Yikan Cheng, Yuan Chen, Zhensen Lin, Xi Zhang, Zhihong Huang, Jiawei Cai, Wenfeng Huang, Yanyun Lin, Haoxian Ke, Jiahui Long, Yifeng Zou, Shubiao Ye, Ping Lan

**Affiliations:** 1 Guangdong Institute of Gastroenterology; Guangdong Provincial Key Laboratory of Colorectal and Pelvic Floor Diseases, The Sixth Affiliated Hospital, Sun Yat-Sen University, Guangzhou, Guangdong People’s Republic of China; 2 Department of Hepatobiliary and Pancreatic Surgery, Sun Yat-Sen University Cancer Center, Guangdong Guangzhou, People’s Republic of China; 3 Department of Radiation Oncology, The Sixth Affiliated Hospital, Sun Yat-Sen University, Guangzhou, Guangdong People’s Republic of China; 4 School of Medicine, Sun Yat-Sen University, Shenzhen, Guangdong People’s Republic of China; 5 Guangzhou Biosyngen Co., Ltd., Guangdong, People’s Republic of China; 6 Department of General Surgery (Department of Colorectal Surgery), The Sixth Affiliated Hospital, Sun Yat-Sen University, Guangdong Guangzhou, People’s Republic of China; 7 Biomedical Innovation Center, The Sixth Affiliated Hospital, Sun Yat-sen University, Guangzhou, People’s Republic of China; 8 State Key Laboratory of Oncology in South China, Sun Yat-sen University, Guangzhou, People’s Republic of China

**Keywords:** IFI35, CD8^+^ T cells, Immunotherapy, Colorectal cancer

## Abstract

**Background:**

A large proportion of the patients with cancer do not respond to immunotherapies. Recent studies suggested an important role for tumor-infiltrating cytotoxic T lymphocytes (CTL) in enhancing response to immunotherapy. Here, we aim to identify gene that induce proliferative and cytotoxic states of CD8^+^ T cells, and to investigate its effect on CAR-T cells against colorectal cancer.

**Methods:**

Correlation between the expression of IFI35 with the activation and cytotoxicity of CD8^+^ T cells was assessed with TCGA and proteomic databases. Then we constructed murine colon cancer cells over-expressing IFI35 and tested their effect on anti-tumor immunity in both immunodeficient and immunocompetent mouse models. Flow cytometry and immunohistochemistry were performed to assess the immune microenvironment. Western blot analysis was used to identify the potential down-stream signaling pathway regulated by IFI35. We further investigated the efficacy of the rhIFI35 protein in combination with immunotherapeutic treatment.

**Results:**

The transcriptional and proteomic analysis of the activation and cytotoxicity of CD8^+^ T cells in human cancer samples demonstrated that IFI35 expression is correlated with increased CD8^+^ T cell infiltration and predicted a better outcome in colorectal cancer. The number and cytotoxicity of CD8^+^ T cells were significantly increased in IFI35-overexpressing tumors. Mechanistically, we identified that the IFNγ-STAT1-IRF7 axis stimulated IFI35 expression, and that IFI35-mediated regulation of CD8^+^ T cell proliferation and cytotoxicity was dependent on PI3K/AKT/mTOR signaling pathway in vitro. Furthermore, IFI35 protein enhanced the efficacy of CAR-T cells against colorectal cancer cells.

**Conclusion:**

Our findings identify IFI35 as a new biomarker that can enhance the proliferation and function of CD8^+^ T cells, as well as increase the efficacy of CAR-T cells against colorectal cancer cells.

**Graphical Abstract:**

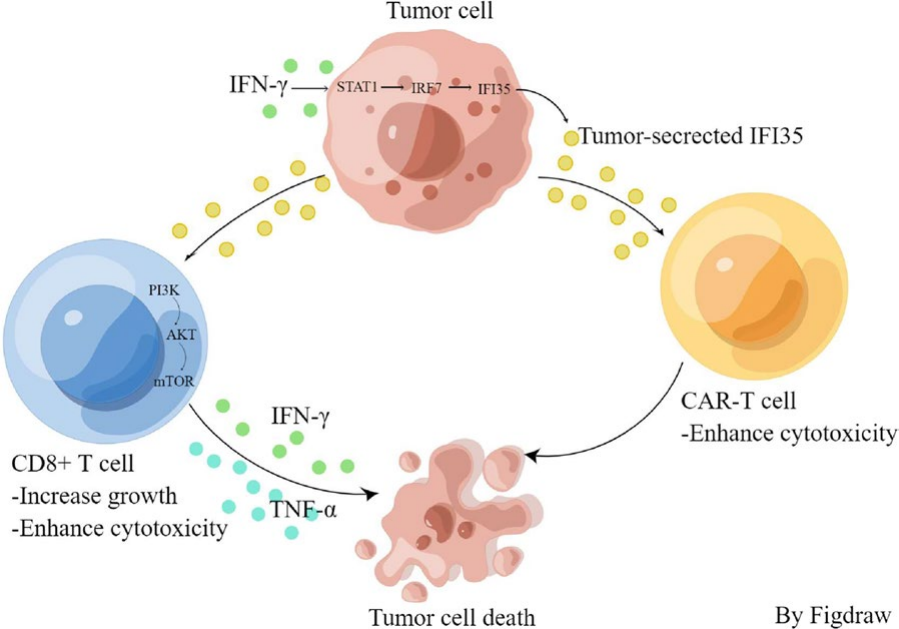

**Supplementary Information:**

The online version contains supplementary material available at 10.1186/s12929-023-00930-6.

## Background

We bear witness the striking breakthroughs in using immune checkpoint inhibitors (ICIs) for diverse cancers, including CRC [1, 2]. Yet the beneficial effect was observed with only a small percentage of patients treated with ICIs [3]. Therefore, there is an urgent need to improve the efficacy of ICI so to benefit more patients and with more tumor types.

In many situations, one of the major causes of non-responsiveness to immune checkpoint blockade therapies is the limited mobilization of tumor-infiltrating cytotoxic T lymphocytes (CTLs) [4–6]. Tumors can be classified as CTL-high and CTL-low based on the number of CD8^+^ T cells, and the CTL-high tumors are more sensitive to immunotherapy than the CTL-low tumors [7]. This is supported by the observation that a key determinant of a durable response to anti-PD-1 therapy was the preexisting T cells in tumor tissue [8]. In addition, CTL was recognized to affect the efficacy of immunotherapy in patients with colorectal cancer [9]. Thus, in this work we aimed to identify the genes that correlate with CTL function and patient outcome with public multi-omics datasets. We found that IFI35 expression positively correlated with the activation of CD8^+^ T cells and with better overall survival in patients with colorectal cancer. This analysis suggested that IFI35 protein may represent a promising novel target for improving immunotherapy sensitivity with colorectal cancer.

Interferon-induced protein-35 kDa (IFI35), widely expressed in epithelial cells, fibroblasts, and monocytes/macrophages, is inducible by both type I and type II interferons [10]. IFI35 is involved in the regulation of virus-related immune inflammatory responses in a varieties of cells and tissues [11–13]. IFI35 has also been reported to promote an inflammatory response in chronic kidney diseases and is associated with renal mesangial cell proliferation in lupus nephritis [14]. Moreover, IFI35 is a DAMP released by lipopolysaccharide (LPS)-activated macrophages to promote inflammatory responses in sepsis [15]. IFI35 is down-regulated in patients with Sezary syndrome and may suppress tumors [16]. A recent study demonstrated that the expression levels of IFI35 influence the radiosensitivity of colorectal cancer cells [17]. However, its role in the adaptive immunity of cancer is unknown. Here, we demonstrated that IFNγ-STAT1-IRF7 axis stimulated-IFI35 directly promoted proliferation and cytotoxicity of CD8^+^ T cells through the PI3K/AKT/mTOR signaling pathway in vitro. Moreover, we showed that the CD8^+^ T cell dependent anti-tumor effect of IFI35 could be explained by its up-regulation of the proliferation and function of CD8^+^ T cells. Our results indicate that IFI35 is promising for enhancing immunotherapeutic response against CRC.

## Methods

### Reagents

Trichloroacetic acids (T104257) was from Aladdin. Poly (I:C) (P9582) was purchased from Sigma Aldrich. Poly (dA:dT) naked (tlrl-patn) and LyoVec™(lyec-12) were purchased from InvivoGen. Human IL-2 (AF-200-02-1000) was from PeproTech. Fludarabine (NSC 118218) was purchased selleck.

### Cell culture

The mouse colon carcinoma cell line, CT26 was maintained in RMPI 1640 medium. The murine colon cancer cell MC38 was cultured in high-glucose Dulbecco’s Modified Eagle Medium. All cell culture medium was supplemented with 10% FBS and 1% penicillin/streptomycin. CT26 cell was from the American Type Culture Collection. MC38 cell was from the China Center for Type Culture Collection. All cell lines were confirmed negative for mycoplasma by PCR-based method.

### Lentiviral production and transduction

The PLKO vector was used to express the shRNAs targeting mouse IFI35. The sequence of the shRNA was 5′-CCGGGCCGAGATCAAATTCCAGCAACTCGAGTTGCTGGAATTTGATCTCGGCTTTTTG-3′. The sequence for mouse IFI35 was amplified from MC38 genomic DNA and cloned into the pCDH-CMV-MCS-EF1-Puro vector using a ClonExpress II One Step Cloning Kit (C112-02, Vazyme). 293T cells were co-transfected with the target plasmid together with packaging plasmids psPAX2 and pMD2.G at a 4:3:1 ratio using PEI reagent as recommended by the manufacturer for the lentivirus package. The viral supernatant was collected 48-h post-transfection, filtered through a 0.45 μm filter, and then added to target cells. After 48 h’ infection with the virus supernatant, the tumor cells were selected with the antibiotic. The efficiency was validated by qPCR and immunoblotting.

### Quantitative PCR analysis

Total RNA was isolated by Trizol-Based Method from cells. RNA was retrotranscribed using the HiScript III-RT SuperMix for qPCR (+gDNA wiper) (Vazyme). Quantitative PCR (qPCR) was performed on cDNA using ChamQ Universal SYBR qPCR Master Mix (Vazyme). The ΔΔCT method was used to calculate fold changes of mRNA using GAPDH or ACTB as an endogenous control. Results are expressed as fold changes by normalizing to the controls. The primers for gene expression analysis were listed in Additional file 2: Table S1.

### Immunoblotting

After washing with cold PBS, cells were lysed in cold RIPA lysis buffer (Beyotime, P0013B) supplemented with 1× protease and phosphatase inhibitor cocktail (MCE). Lysates were shaken slowly on ice for half an hour and centrifuged at 15,000×*g* for 20 min at 4 °C. A BCA protein assay kit (ThermoFisher) was used to quantify protein concentration. Protein was denatured at 98 °C for 5 min. 10 μg total protein was separated by SDS-PAGE and transferred to a 0.45 µm PVDF membrane (Millipore). The PVDF membranes were incubated with primary antibodies overnight at 4 °C after incubating with 5% w/v bovine serum albumin (BSA) for 1 h. The next day the membrane was hybridized with an HRP-conjugated secondary antibody (Promega) for 1 h at room temperature. Protein bands were detected using Western ECL Blotting Substrates (Bio-Rad) and captured using ChemiDoc™ Imaging System (Bio-Rad). The primary antibodies used include: anti-Ifi35 (#HPA045946, Sigma-Aldrich), anti-PI3K (#4292, CST), anti-p-PI3K (#AF324, Affinity), anti-mTOR (#2972, CST), anti-p-mTOR (#5536, CST), anti-AKT (#9272, CST), anti-p-AKT (#9271, CST), anti-ERK1/2 (#4695, CST), anti-p-ERK1/2 (#4370, CST), anti-JNK (#9252, CST), anti-p-JNK (#4668, CST), anti-P38 (#8690, CST), anti-p-P38 (#4511, CST), anti-P65 (#10745-1-AP, Proteintech), anti-p-P65 (#3033, CST), anti-stat3 (#4904, CST), anti-p-stat3 (#9145, CST), anti-GAPDH (#10494-1-AP, Proteintech), and anti-Tubulin (#11224-1-AP, Proteintech).

### In vitro proliferation assay of murine colon cancer cells

For cell proliferation assay, cells were seeded in flat-bottom 96 well plates at a density of 3000 cells/well. Cell proliferating rate was determined by counting the cells with the counting kit-8 (APExBIO) at 0, 24, 48, and 72 h.

### Animal experiments

Five to 6-week-old female C57bl/6 mice, male BALB/c, and nude mice were purchased from GemPharmatech (Nanjing, China). Mice were maintained at a specific pathogen-free facility with a 12 h/12 h day/night turnover. All animal experiments were approved by the Research Ethical Committee of the Sixth Affiliated Hospital of Sun Yet-sen University and in accordance with the National Institutes of Health Guide for the Care and Use of Laboratory animals. For the subcutaneous mouse model, 2 × 10^5^ MC38 or 1 × 10^5^ CT26 with IFI35 stable knockdown or IFI35 overexpression constructs, or control cells suspended in 0.1 mL PBS were injected into the flanks of the mice. Tumor growth was assessed by caliper measurement three times in 1 week. The following formula was used to calculate tumor volume: Tumor volume (mm^3^) = (tumor width^2^ × length)/2. On day 7 after tumor cell injection, anti-PD-1 (InVivoMab) or IgG1 isotype monoclonal antibodies (InVivoMab) were intraperitoneally injected at a dose of 200 µg per mouse every 3 days for the duration of the experiment.

### Fluorescent immunohistochemistry

Fluorescent Immunohistochemistry was performed using a PANO IHC kit (Panovue, Beijing, China). After anti-Ifi35 (#HPA045946, Sigma-Aldrich) was applied, followed by horseradish peroxidase (HRP)-conjugated secondary antibody incubation and tyramide signal amplification. Nuclei were stained with 4′-6′-diamidino-2-phenylindole (DAPI, Abcam) after all antigens had been labeled. Then, the stained slides were scanned using the Mantra System (PerkinElmer, Waltham, Massachusetts, US) to obtain multispectral images. The cells that were CK positive and had malignant cytomorphology were recognized as tumor cells. Tumor cells were further confirmed with reference to HE-stained slides. inForm software was used to determine H-score of the expression of IFI35 in tumor.

### Immunohistochemistry

Sections from paraffin-embedded murine tissue of CT26 or MC38 colon adenoma samples were deparaffinized twice in xylene and then hydrated through graded concentrations of ethanol. Antigen retrieval were performed in Citrate Antigen Retrieval solution (Zhong Shan Jin Qiao) in a microwave oven. Endogenous peroxidase activity was eliminated by 3% Hydrogen peroxide. The sections were blocked with Normal Goat Serum (Zhong Shan Jin Qiao) and then incubated with anti-CD8 (Affinit, #AF5126) overnight at 4 °C. Then the slide was incubated at 37 °C for 1 h with a secondary antibody and was developed with a DAB kit. Images were captured using a camera. To quantify the signals, 5 fields were randomly selected from each sample, and examined by 2 pathologists in a double-blind manner.

### Isolation of tumor single cells

The excised murine tumors were dissociated and digested for 1 h at 37 °C in a digestion medium composed of 20 µg/mL DNase I (Gibco), and 5 µg/mL Collagenase IV (Gibco) in RPMI medium and filtered using 70 µm Cell Strainer (Falcon). Single-cell suspensions were then treated with red blood cell lysis buffer (Invitrogen), washed and resuspended with PBS.

### Flow cytometry

Tumor single cells were incubated with anti-CD16/32 antibodies (eBioscience) for 20 min on ice. For surface staining, cells were incubated with the dilution of the surface marker antibodies for 30 min and were washed in FACS buffer. Cells were then directly analyzed by flow cytometry. For cytokine staining, cells were stimulated for 6 h with ionomycin (MCE) and phorbol 12-myristate13-acetate (MCE) in the presence of monensin (MCE) and Brefeldin A (MCE) and stained for surface molecules followed by treatment with IC fixation buffer (ThermoFisher). Then the cells were incubated with specific cytokine antibodies for another 30 min on ice. According to the manufacturer’s instructions, the Foxp3/Transcription Factor Staining Buffer Set (Invitrogen) was used to detect nucleus protein FOXP3. CytoFLEX flow cytometer (Beckman Coulter) was used to acquire and analyze all events. The following antibodies were used: FITC anti-CD45 (103108, BioLegend), Brilliant Violet 510 anti-CD45 (103138, Biolegend), PE/Cyanine7 anti-CD11c (117317, BioLegend), APC anti-CD11b (101212, BioLegend), PerCP-Cyanine5.5 anti-CD11b (45-0112-80, eBioscience),Brilliant Violet 605 anti-Ly-6G (127639, BioLegend), PE anti-Ly-6C (12-5932-80, eBioscience), PE anti-IFNγ (505808, BioLegend), APC anti-IFNγ (17-7311-81, Invitrogen), PE anti-TNFα (12-7321-81, eBioscience), APC anti-TNFα (17-7321-81, Invitrogen), PerCP-Cyanine5.5 anti-CD8 (45-0081-82, eBioscience), APC anti-F480 (123115, eBioscience), Brilliant Violet 421 anti-mouse CD335 (NKp46) (137612, BioLegend), PE anti-CD19 (115507, BioLegend), APC anti-TCR γ/δ (118116, Biolegend), PE anti-CD4 (12-0041-82, Invitrogen), APC anti-FOXP3 (17-5773-80, eBioscience), PE anti-CD206 (141705, BioLegend), APC Anti-CD86 (105012, BioLegend), Brilliant Violet 421 anti-PD1 (135218, BioLegend), eFluorTM 660 anti-TOX (50-6502-82, eBioscience), PE anti-LAG3 (125208, BioLegend), PE anti-Ki67 (12-5698-82, eBioscience), PE anti-CD39 (12-0391-82, eBioscience) and Fixable Viability Dye eFluor™ 780 (65-0865-14, eBioscience).

### Enzyme-linked immunosorbent assay

The cell culture medium of the murine colon cancer cells was replaced at 80% confluency with a complete medium containing 2% FBS. After 48 h, the cell culture supernatant was collected to eliminate debris by centrifugalization and concentrated using Millipore (MERCK). Secretion of IFI35 protein into the culture supernatant was detected with Mouse Ifi35 ELISA Kit (E10460m) according to manufacturer’s instructions.

### Carboxyfluorescein succinimidyl ester proliferation assay

EasySep™ Mouse CD8^+^ T Cell Isolation Kit (Stemcell) was used to isolate mouse CD8^+^ T cell from the spleen. Isolated CD8^+^ T cells were labeled with 2 µmol/L carboxyfluorescein succinimidyl ester (Invitrogen) at a concentration of 106 cells/mL in PBS at 37 °C for 15 min. CSFE-labeled CD8^+^ T cells were cultured with the tumor supernatants in 96-well round-bottom plates pre-coated with anti-CD3 (1 µg/mL), anti-CD28 (1 µg/mL), and IL-2 (10 ng/mL) at 4 °C overnight. After 3 days, the proliferation of CD8^+^ T cells was assessed by measuring CFSE dilution using the CytoFLEX flow cytometer.

### In vitro T cell migration assay

A polycarbonate membrane with a 5.0 μm pore size (Oxygens) was used to perform migration assay. CD8^+^ T cells activated with anti-CD3 (1 µg/mL), anti-CD28 (1 µg/mL), and IL-2 (10 ng/mL) were washed twice, resuspended in 100 µL serum-free culture medium, and then added to the top chamber. The concentrated supernatant from differently treated colon cancer cells was added to the bottom chamber. After 12 h of culture, the CD8^+^ T cells at the bottom of the chamber were collected and quantitated by FACS.

### Annexin V/propidium iodide (PI) assay

For apoptosis assays, cells were stained with an Annexin V/7-Amino Actinomycin D (Annexin V/7-AAD) apoptotic Kit (BD Pharmingen) at 4 °C in the dark for 15 min, and the apoptotic cells were examined by FACS.

### OT-I cells isolation and co-culture with OVA^+^ tumor cells

Spleen cells from OT-I mice were isolated with EasySep™ Mouse CD8^+^ T Cell Isolation Kit and then stimulated with anti-CD3 (1 µg/mL), anti-CD28 (1 µg/mL), and IL-2 (10 ng/mL) for 2 days. Then the OT-I cells were cocultured with OVA^+^ tumor cells for 8 h at a 1:1 ratio and harvested for flow cytometry analysis.

### Generation of CEA CAR-T cells

Briefly, the production of the CEA CAR lentiviral vector and the transduction of human T cells were carried out according to a previous study [18] with several modifications. The CEA CAR molecule consists of a CD8 signal peptide, an anti‐CEA scFv, a hinge region and a transmembrane domain from CD8, and the cytoplasm domains 4-1BB and CD3ζ. The codon-optimized sequence of the CEA CAR was synthesized by GENEWIZ and cloned into the pCDH-EF1α vector, resulting in the plasmid pCDH-EF1α-CEA CAR. The lentiviral vector was generated by co-transfection of pCDH-EF1α-CEA CAR along with pMD2.G, pMDLg/pRRE, and pRSV-Rev into 293T cells with Lipofectamine 3000 (Invitrogen) according to the manufacturer’s manual. At 48 h post transfection, the lentiviral vector was harvested and filtered with 0.45-μm membrane and concentrated with the Lenti-X concentrator (Takara Bio, San Jose, CA, USA). For transduction of T cells, human PBMC was stimulated with CD3 and CD28 antibodies (Biogems, Westlake Village, CA, USA) for 24 h, and the lentiviral vector from above was added at an MOI of 5 IU/cell. The culture medium was replaced at 72 h post transduction and the CAR-T cells were harvested, analyzed with flow cytometry, subjected for downstream analysis, or stored in liquid nitrogen at 10 days post transduction.

### CAR T-cell cytotoxicity assays

10^6^ CAR T cells were incubated with LOVO tumor cells at an E/T of 10:1 in the absence or presence of rhIFI35. After 5 h, all cells in the well were collected and washed with PBS and analyzed by flow cytometry.

### Statistical analysis

Statistical analysis was performed using GraphPad Prism statistical software (version 7, GraphPad Software Inc.) and statistical significance was defined as a p-value less than 0.05. Tumor growth was analyzed using two-way ANOVA. Survival functions were estimated using the Kaplan–Meier method. In other experiments, comparisons between two groups were conducted by unpaired two-sided Student’s t-tests.

## Results

### Tumor IFI35 correlates with CD8^+^ T cells expression and patient outcome

**Fig. 1:**
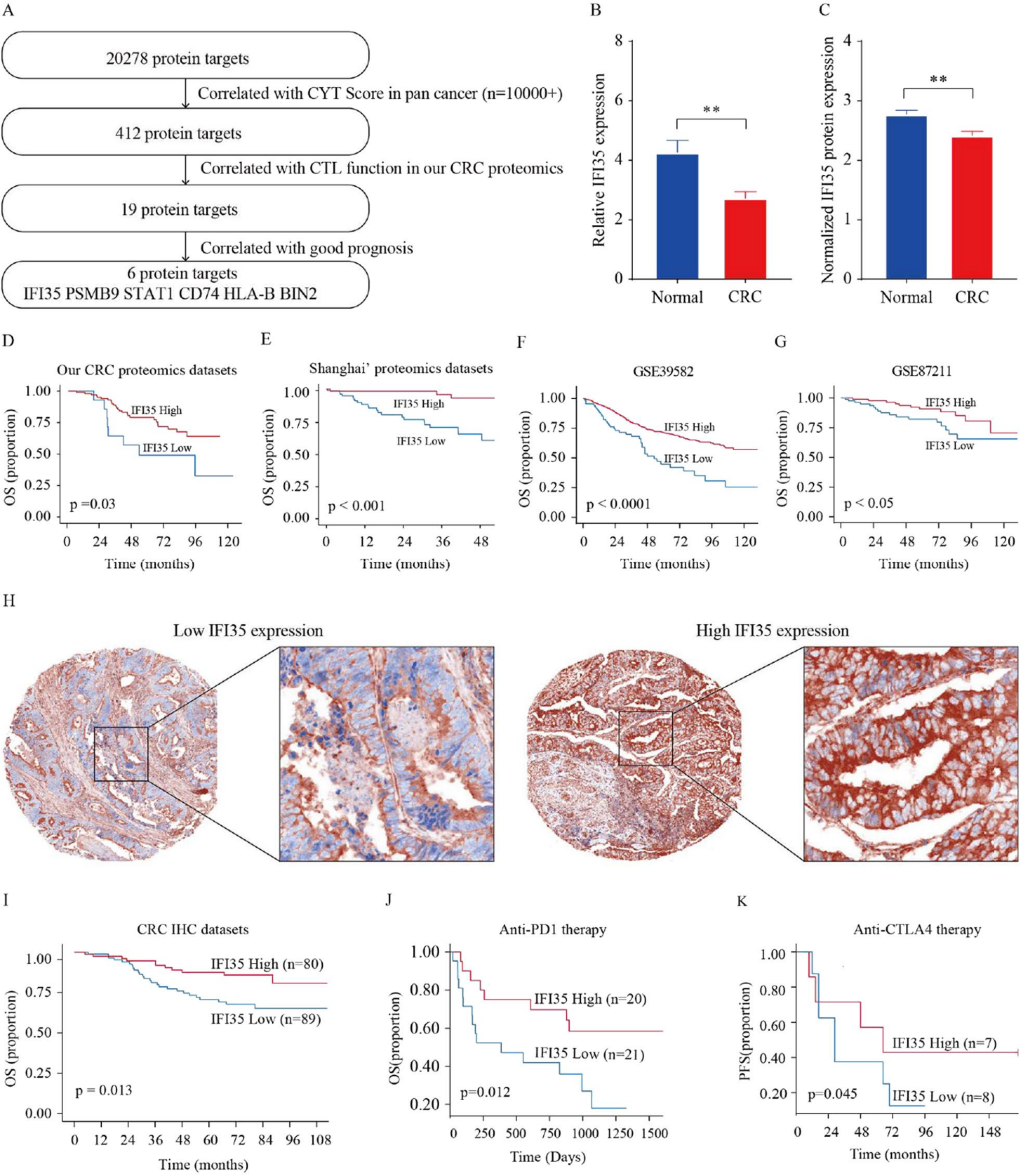
Tumor IFI35 correlates with CD8^+^ T cells expression and patient outcome. **A** Screening of CTL function-correlated genes associated with good prognosis in human colorectal cancer. In the TCGA dataset, 412 top genes were identified as positively correlated with Cytolytic Activity (CYT) Score in pan-cancer using Spearman correlation analysis. Among the shared 412 genes, based on our own CRC proteomics, 19 proteins were identified to be correlated with CTL function signature on the protein level using Spearman correlation analysis. The proteomic analysis detected 6 out of 19 proteins with a good prognosis in CRC through Kaplan–Meier analysis. **B**, **C** IFI35 expression in normal colorectal tissues and colorectal cancer tissues. **B** Statistical analysis of IFI35 mRNA levels of 66 paired samples of CRC and adjacent normal tissues from the Sixth Affiliated Hospital (n = 66, non-parametric Wilcoxon matched-pairs signed rank test, *p* < 0.01). **C** IFI35 protein expression in colorectal adenocarcinoma tissues (n = 90) and normal colorectal tissues (n = 30). Two tailed t-tests, *p* < 0. 01. **D**–**G** Kaplan–Meier analyses of the association of overall survival with IFI35 proteins. **D** Overall survival analysis in CRC with low (n = 14) and high (n = 101) levels of IFI35 in 115 CRC patients from our CRC proteomics datasets, **E** overall survival analysis in CRC with low (n = 81) and high (n = 64) levels of IFI35 in 145 CRC patients from Shanghai’ proteomics datasets, **F** overall survival analysis in CRC with low (n = 69) and high (n = 487) levels of IFI35 in 556 CRC patients from GSE39582, **G** overall survival analysis in CRC with low (n = 85) and high (n = 105) levels of IFI35 in 190 CRC patients from GSE87211. Log-rank test. The optimal cutoff values were calculated by the X-tile software. **H**, **I** Expression of IFI35 protein and its prognostic value in CRC. **H** The expression of IFI35 in CRC was detected by Fluorescent Immunohistochemistry analysis based on tissue microarray. **I** Patients with high expression of IFI35 (n = 80) showed better overall survival time than the patients with low expression of IFI35 (n = 89). **J**, **K** Relationship between IFI35 expression and immunotherapy efficacy in melanoma patients. **J** Overall survival analysis in melanoma with low (n = 21) and high (n = 20) levels of IFI35 treated with Anti-PD1 therapy. **K** Progression-free survival was analyzed and compared between patients with low (n = 8) and high (n = 7) levels of IFI35 in melanoma patients treated with Anti-CTLA4 therapy. Log-rank test

We initially screened the pan-cancer dataset in The Cancer Genome Atlas (TCGA) and selected 412 genes correlated with Cytolytic Activity (CYT) Score using the ssGSEA algorithm [19]. We further tested these 412 genes with our colorectal proteomics dataset and uncovered 19 proteins positively correlated with cytotoxicity of CD8^+^ T cells using the ssGSEA algorithm. Among these 19 proteins, further analysis identified six proteins (including IFI35, PSMB9, STAT1, CD74, HLA-B, and BIN2) that were associated with a favorable prognosis in colorectal cancer (Fig. 1A). Among them, HLA-B and STAT1 are well-known proteins associated with anti-tumor immunity. IFI35, a secreted protein that acts as DAMPs to promote host inflammatory responses in sepsis [15], caught our attention as it has been identified as a mature functional protein and a potential therapeutic candidate for cancer treatment. Therefore, we shifted our research focus to IFI35. Again with our proteomic datasets, we observed that IFI35 expression was positively correlated with T cell activation and cytotoxicity [20, 21] (Additional file 1: Fig. S1A, B). Further, with the pan-cancer datasets in TCGA, the IFI35 mRNA levels were positively correlated with the CD8^+^ T cells (Additional file 1: Fig. S1C–H).

First, we detected mRNA levels of IFI35 in normal tissues were significantly higher than the match colorectal cancer tissues (Fig. 1B). Additionally, using proteomic analysis from a previous study that employed LC–MS/MS and tandem mass tagging [22], we investigated IFI35 protein expression levels, and our findings showed that IFI35 was significantly upregulated in normal colon tissues compared to colon cancer tissues (Fig. 1C). Next, we examined the prognostic role of IFI35 in CRC and found that high expression of IFI35 predicted a good prognosis with our CRC proteomics dataset, Shanghai’ proteomics datasets, and multiple GSE databases (Fig. 1D–G). To validate these results, we conducted immunohistochemistry staining with a new cohort including 169 patients with CRC. We found that low expression of IFI35 in cancer tissue was associated with poor prognosis in patients with colorectal cancer (Fig. 1H, I). We extended our studies from colorectal cancer to other types of cancers. We found that IFI35 expression was positively correlated with patients’ survival (Additional file 1: Fig. S1I–K). Together, these findings suggest that IFI35 is a tumor suppressor in CRC.

To explore the potential value of IFI35 in cancer immunotherapy, we examined the impact of IFI35 expression levels on immunotherapy efficacy on a web platform TIDE [7, 23]. Our analyses demonstrated that patients with high IFI35 expression levels were sensitive to immunotherapy, which suggests that IFI35 is a novel factor determining clinical response to immunotherapy (Fig. 1J, K) [24, 25]. Collectively, high expression level of IFI35 was positively correlated with CD8^+^ T cells, a good prognosis and higher efficacy in immunotherapy with cancer patients.

### IFI35 plays an essential role in tumor growth in an immune-dependent way

**Fig. 2:**
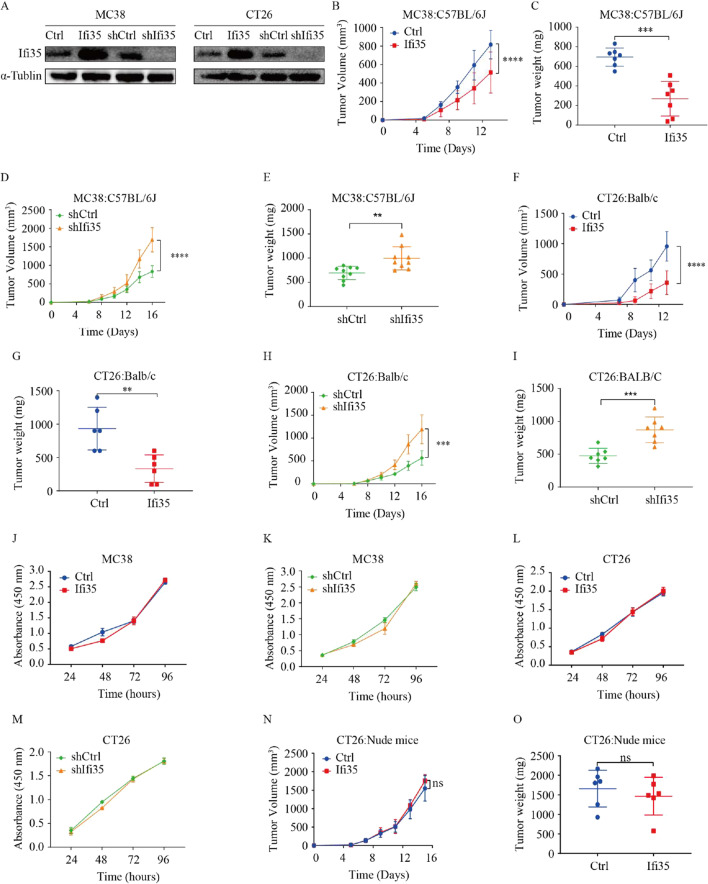
IFI35 plays an important role in tumor growth in an immune-dependent way. **A** Western blot analysis of MC38 and CT26 cells transfected with plasmid harboring mIFI35, pCDH-mIFI35 or shRNA targeting mIFI35, pLKO-mIFI35 plasmids with IFI35 antibody. **B**–**I** Effect of IFI35 on murine colorectal tumor growth rate and tumor weight. **B**, **C** The tumor size and tumor weight of C57BL/6 mice injected subcutaneously with vector control and IFI35-overexpressing MC38 cell line. n = 7 mice for both groups. **D**, **E** The tumor size and tumor weight of C57BL/6 mice bearing scramble and sh-IFI35 expressing MC38 cells. n = 9 mice for both groups. **F**, **G** The tumor size and tumor weight of vector control and IFI35-overexpressing CT26 cell line in Balb/c mice. n = 6 mice for both groups. **H**, **I** Tumor growth in syngeneic wild-type mice bearing scramble and sh-IFI35 expressing CT26 cells. n = 7 mice for both groups. Values are represented as mean ± SD. P values calculated by two-way ANOVA in **B**, **D**, **F**, **H**. Tumor weight was determined by two tailed t-tests in **C**, **E**, **G**, **I**. **P < 0.001, ***P < 0.0005, ****P < 0.0001. **J**–**M** Cell growth of IFI35-knockdown or IFI35-overexpressing MC38 (**J, K**) and CT26 (**L, M**) cells in vitro. n = 3. Values are represented as mean ± SD. Two-way ANOVA, ns: not significant. **N**, **O** Growth rate (**N**) and endpoint tumor weight (**O**) of vector control and IFI35Kd CT26 tumors. In each case, about 2 × 10^5^ tumor cells were injected subcutaneously and observed for tumor formation in nude mice. n = 6 mice for each group. Values are represented as mean ± SD. P values calculated by two-way ANOVA in **N** and by two tailed t-tests in **O**, *ns* not significant

The above results suggest that IFI35 may play a role in tumor immunity. To test this hypothesis, we performed IFI35 knock-down study using a short hairpin RNA against IFI35, and IFI35 over expression study with an IFI35 expression plasmid in murine CT26 and MC38 colon cancer cells. Stable cell lines were constructed and confirmed by western blots (Fig. 2A). We then injected murine CT26 and MC38 colon cancer cells into syngeneic immunocompetent BALB/C and C57BL/6 mice subcutaneously to investigate the role of IFI35 in antitumor immunity. In mice inoculated with shIFI35 cells, IFI35 knockdown significantly promoted tumor growth measured by tumor volume and weight in both murine colon cancer models. Moreover, IFI35-overexpressing tumor cells caused slower tumor growth in both mouse models (Fig. 2B–I). Next, we wish to exclude the possibility that IFI35 has a direct impact on the proliferation of colorectal cancer cells. Our results showed that neither downregulation nor upregulation of IFI35 altered the growth rates of mouse CRC cells in vitro (Fig. 2J–M). On the other hand, with nude mice inoculated with CT26 cells overexpressing IFI35, similar rates of tumor growth were observed compared with nude mice bearing control CT26 colon tumor cells (Fig. 2N, O). These in vitro and in vivo data suggested that the impact of IFI35 on tumor growth likely involves an immune-dependent mechanism.

### IFI35 increases the intratumoral number of CD8^+^ T cells

**Fig. 3:**
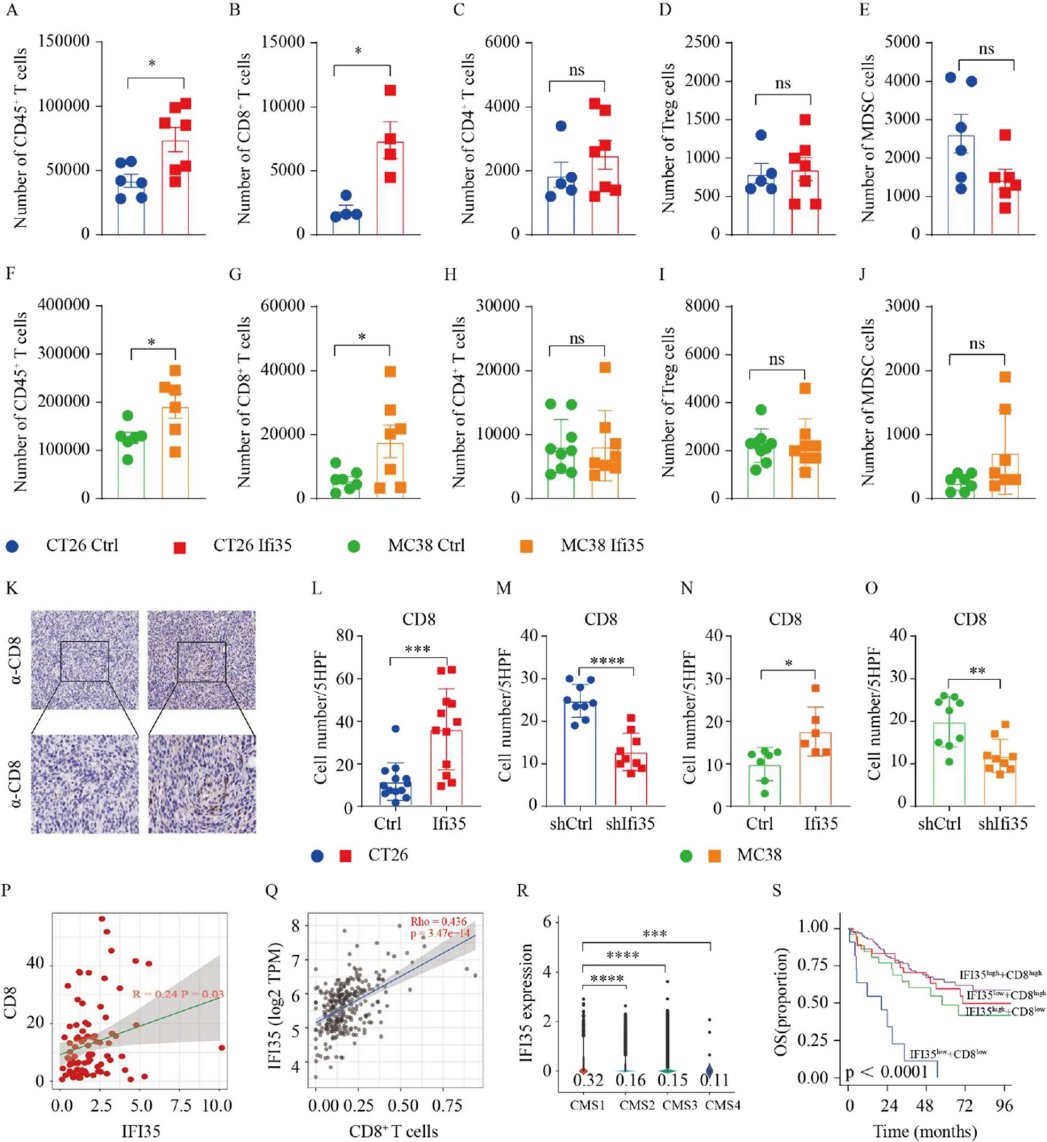
IFI35 increases the intratumoral number of CD8^+^ T cells. **A**–**E** Effect of IFI35 overexpression on CT26 tumor-infiltrating various immune cells. IFI35 transfected CT26 cells were inoculated into BALB/c mice. The numbers of tumor-infiltrating CD45^+^ T cells (**A**), CD8^+^ T cells (**B**), CD4^+^ T cells (**C**), Foxp3^+^ cells (**D**), and MDSC cells (**E**) were analyzed by flow cytometry. Cell numbers are given for 1000,000 total cells from each tumor. Values are represented as mean ± SEM. P values were determined by two tailed t-tests. *P < 0.05, **P < 0.01, *ns* not significant. **F**–**J** Effect of IFI35 overexpression on MC38 tumor-infiltrating various immune cells. IFI35 transfected MC38 cells were inoculated into C57/BL6 mice. The numbers of tumor-infiltrating CD45^+^ T cells (**F**), CD8^+^ T cells (**G**), CD4^+^ T cells (**H**), Foxp3^+^ cells (**I**), and MDSC cells (**J**) were analyzed by flow cytometry. Cell numbers are given for 1000,000 total cells from each tumor. Values are represented as mean ± SEM. P values were determined by two tailed t-tests. *P < 0.05, **P < 0.01, *ns* not significant. **K**–**O** Quantification of CD8^+^ T cells from paraffin sections of CT26 and MC38 colon cancer by IHC. **K** Representative IHC staining for CD8 in paraffin sections of MC38 colon cancer. **L**–**O** Average cell number per high-power field (HPF) is shown; Two tailed t-tests, *P < 0.05, **P < 0.01, ***P < 0.001, ****P < 0.0001. **P** Positive correlation of IFI35 protein levels with that of CD8 IHC levels in human colorectal cancer. **Q** Scatterplots showing correlation between IFI35 expression and CD8^+^ T cell expression level in The Cancer Genome Atlas Colon Adenocarcinoma (TCGA-COAD) data collection determined by tumor immune estimation resource (Timer, http://cistrome.dfci. harvard.edu/TIMER/) website. **R** Violin plot showing expression of IFI35 between four CMS subtypes in the single-cell cohort. **S** Kaplan–Meier analysis of overall survival (OS) in CRC patients with low IFI35 and low CD8 expression (n = 11), low IFI35 and high CD8 expression (n = 37), high IFI35 and low CD8 expression (n = 26), high IFI35 and high CD8 expression (n = 103). The corresponding cutoff values was calculated by the maxstat algorithm in R package “maxstat”

To explore the immune mechanism by which IFI35 may affect tumor growth, we analyzed the immune cell composition in control and IFI35-overexpressing murine tumors by flow cytometry. The gating strategies used to identify and quantity different immune cell subtypes that infiltrated tumors are presented in Additional file 1: Fig. S2A. Flow cytometry analysis showed that IFI35-overexpressing CT26 tumors have more CD45^+^ T cells, effector NK cells, and CD8^+^ T effector cells, while showing decreased numbers of DC cells as compared to control tumors. However, no significant differences were observed in CD4^+^ T cells, Treg cells, γδT cells, B cells, MDSC cells, and MΦ cells (Fig. 3A–E, Additional file 1: Fig. S2B–F). Furthermore, tumors bearing IFI35-overexpressing MC38 cells showed an increase in CD45^+^ T cells, CD8^+^ T cells, when compared with tumors bearing control cells (Fig. 3F–J, Additional file 1: Fig. S2G–K).

We also conducted functional evaluations of macrophages and Treg cells, both of which play crucial roles in regulating the immune response. Tumor-associated macrophages (TAMs) can be polarized towards pro-inflammatory M1 macrophages or immune-suppressive M2 macrophages [26, 27]. The gating strategies employed to identify TAMs and exhaustion markers on Treg cells are illustrated in Additional file 1: Fig. S3A. Our results showed no significant difference in the proportions of M1 and M2 macrophages in the TME of BALB/c mice injected subcutaneously with vector control and IFI35-overexpressing CT26 cell line (Additional file 1: Fig. S3B, C). Furthermore, we measured the expression of well-known immune checkpoint molecules, including lymphocyte activation gene‐3 (LAG‐3), CD39, and cytotoxic T-lymphocyte-associated protein 4 (CTLA‐4) among Treg cells. We found no significant difference in the proportions of exhaustion markers on Treg cells in the TME of BALB/c mice injected subcutaneously with vector control and IFI35-overexpressing CT26 cell line (Additional file 1: Fig. S3D–F).

These results were consistent with our bioinformatics analysis in Additional file 1: Fig. S1 that overexpression of IFI35 increased the enrichment of CD8^+^ T cells, which was the only common up-regulated immune cell subtype in both mouse models. We next performed immunohistochemical staining of tumor tissue to examine the effect of IFI35 overexpression on the number of CD8^+^ T cells. Consistent with flow cytometry, the number of CD8^+^ T cells was increased in tumors injected with IFI35-overexpressing cells compared with control tumors in both murine models. In line with this, loss of IFI35 led to lower tumor number of CD8^+^ T cells (Fig. 3K–O).

Omics data from different resources were examined to further validate the IFI35 role in CD8^+^ T cell expression in CRC. Our proteomic data showed that the protein abundance of CD8 was positively correlated to that of IFI35 protein in samples from patients with colorectal cancer (Fig. 3P). Consistently, IFI35 transcripts correlated with CD8^+^ T cells density with colorectal cancer samples in the TCGA-COAD dataset, as analyzed by the Timer website (Fig. 3Q). CRC could be divided into 4 subtypes according to consensus molecular subtype (CMS), including CMS1 (immune), CMS2 (canonical), CMS3 (metabolic), and CMS4 (mesenchymal), when CMS1 showed high infiltration of CD8^+^ T cells [28, 29]. Therefore we further analyzed the expression of IFI35 in tumor cells from each CMS phenotype. Patients of CMS1 subtype tended to express higher levels of IFI35 than the other three subtypes (Fig. 3R) [30]. Moreover, patients with higher IFI35 and higher CD8 expression showed increased overall survival in another CRC cohort (GSE17536) (Fig. 3S). Together, our data demonstrated that higher IFI35 expression increased the number of CD8^+^ T in colorectal cancer.

### Tumor-secreted IFI35 promotes proliferation and cytotoxic activity of CD8^+^ T cells, and decreased exhausted CD8^+^ T cells

**Fig. 4:**
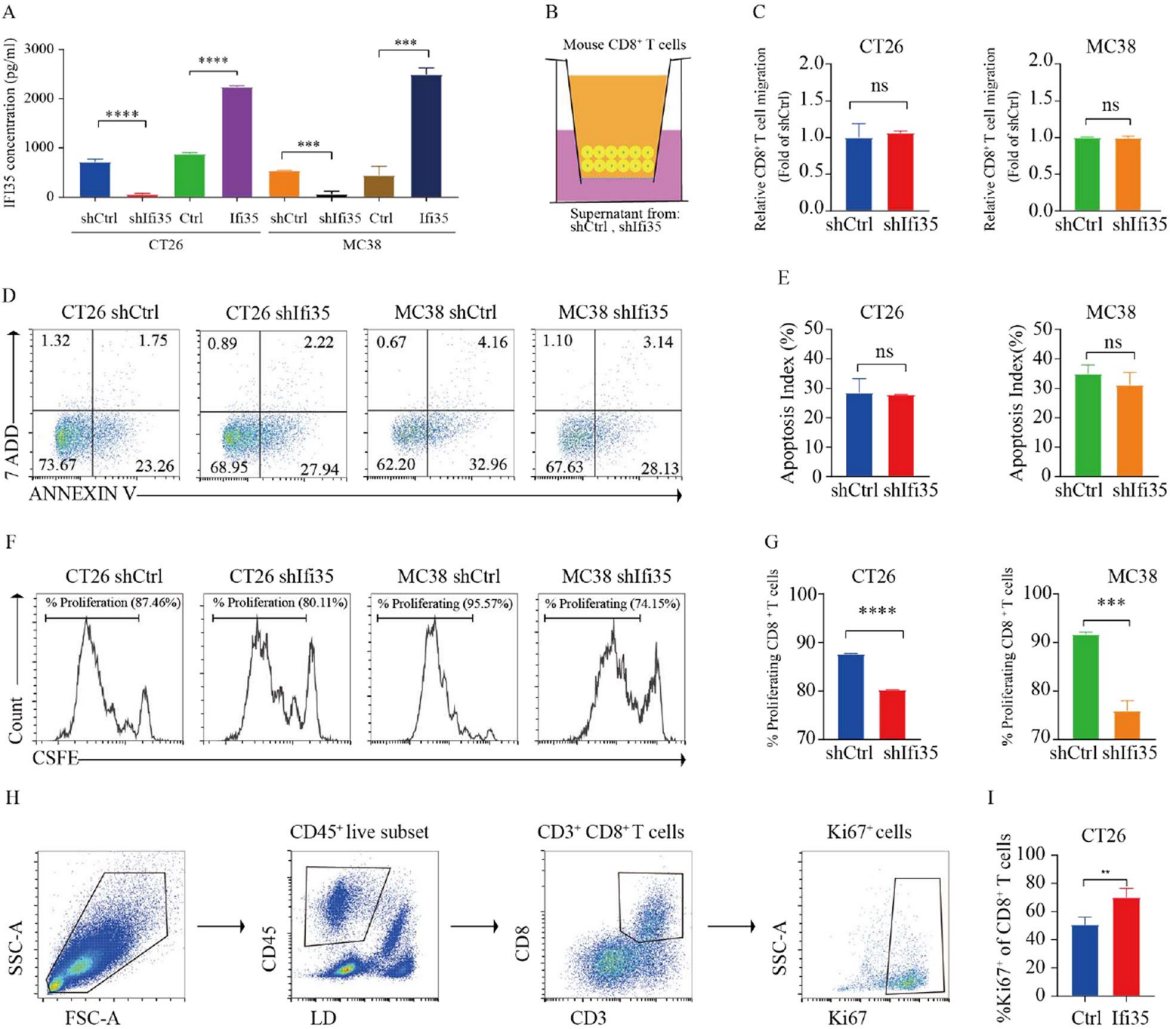
Tumor-secreted IFI35 promotes CD8^+^ T cells proliferation. **A** ELISA analysis in the supernatant of IFI35 overexpressed or knocked down CT26 and MC38 cells. **B**–**G** Effect of IFI35 protein on T cell migration, apoptosis and proliferation in vitro. T cell migration, apoptosis and proliferation in the presence of supernatant from murine colon cancer cells expressing shRNA against IFI35 (shIFI35) and scrambled sequence control (shRNA) for 72 h. In all experiments, mouse splenic CD8^+^ T cells were stimulated with plate-bound anti-CD3/CD28 mAbs. **B**, **C** Migration of CD8^+^ T cells by flow cytometric analysis. **D**, **E** Flow cytometric analysis of mouse splenic CD8^+^ T cells apoptosis. **F**, **G** Proliferation of CFSE-labeled mouse splenic CD8^+^ T cells by flow cytometric analysis. n = 3. Error bars represent the mean ± SEM. Two tailed t-tests, *ns* not significant. ***P < 0.001, ****P < 0.0001. **H**, **I** Effect of IFI35 on T cell proliferation in vivo. **H** Gating strategy for Ki67^+^ CD8^+^ T cells in CT26 tumors. **I** Quantification of Ki67 expression among CD8^+^ T cells in CT26 tumors. n = 4 for both groups. Two tailed t-tests. **P < 0.01

Because IFI35 can be released by activated macrophages [15], we hypothesized that IFI35 is secreted by colon cancer cells to influence nearby CD8^+^ T cells. To test this hypothesis, the culture supernatant of murine colon cancer cells was collected. Enzyme-Linked Immunosorbent Assay (ELISA) result showed that the level of IFI35 was significantly higher in the supernatant of IFI35-overexpressing cells, and lower in the sh-IFI35 cells, compared to control cells in both murine colon cancer cell models (Fig. 4A). Next, CD8^+^ T cells were treated with the supernatants from both murine colon cancer cells expressing shRNA against IFI35 (shIFI35) and scrambled sequence control (shRNA), respectively. No difference in CD8^+^ T cell migration (Fig. 4B, C) or apoptosis (Fig. 4D, E) were observed. However, we found that the proliferation of CD8^+^ T cells was reduced when treated with supernatant from shIFI35 cells as demonstrated by flow cytometry (Fig. 4F, G). To study the effect of IFI35 on CD8^+^ T cells proliferation in vivo we assayed markers indicative of T cell proliferation. CD8^+^ T cells from tumors bearing IFI35-overexpressing CT26 cells were more proliferative, based on Ki67 levels (Fig. 4H, I). These results indicated that tumor-secreted IFI35 directly promoted CD8^+^ T cell proliferation.

**Fig. 5:**
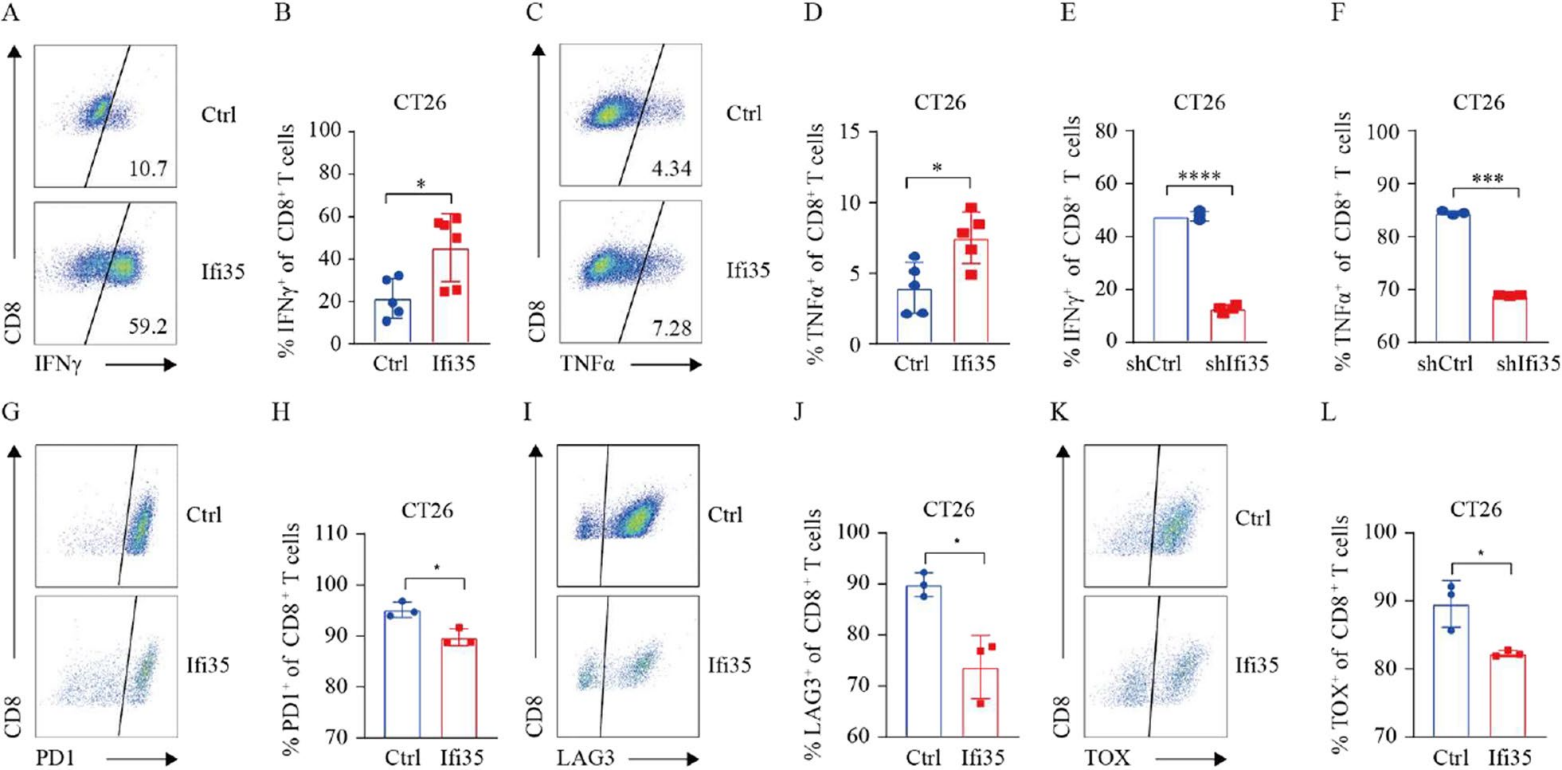
IFI35 promotes CD8^+^ T cell cytotoxicity and alleviates exhaustion. **A**, **B** Representative FACS plots (**A**) and quantification (**B**) of IFNγ expression among CD8^+^ T cells in CT26 tumor. Two tailed t-tests, *P < 0.05. **C**, **D** Representative FACS plots (**C**) and quantification (**D**) of TNFα expression among CD8^+^ T cells in CT26 tumor. Two tailed t-tests, *P < 0.05. **E**, **F** Effect of tumor-secreted IFI35 protein on CD8^+^ T cell effector cytokines. Activated mouse CD8^+^ T cells were cultured in the presence of supernatant from murine colon cancer cells expressing shRNA against IFI35 (shIFI35) and scrambled sequence control (shRNA) for 72 h. IFNγ and TNFα of CD8^+^ T cells were calculated by Flow cytometric analysis. n = 3. Error bars represent the mean ± SEM. Two tailed t-tests, ***P < 0.001, ****P < 0.0001. **G**, **H** Representative FACS plots (**G**) and quantification (**H**) of PD1 expression among CD8^+^ T cells in CT26 tumor. Two tailed t-tests, *P < 0.05. **I**, **J** Representative FACS plots (**I**) and quantification (**J**) of LAG3 expression among CD8^+^ T cells in CT26 tumor. Two tailed t-tests, *P < 0.05. **K**, **L** Representative FACS plots (**K**) and quantification (**L**) of TOX expression among CD8^+^ T cells in CT26 tumor. Two tailed t-tests, *P < 0.05

To explore the impact of IFI35 on CD8^+^ T cell activity, we analyzed the expression of IFNγ and TNFα released by effector CD8^+^ T cells by flow cytometry. Strikingly, we observed that overexpression of IFI35 promoted CD8^+^ T cells to secrete more IFNγ and TNFα in CT26 tumor (Fig. 5A–D). And the levels of effector molecules (IFNγ and TNFα) of CD8^+^ T cells were reduced when treated with supernatant from shIFI35 cells in vitro (Fig. 5E, F). We also examined co-inhibitory receptors of CD8^+^ T in vivo. Accordingly, we found that fewer CD8^+^ T cells expressed PD1, LAG3 and TOX with tumors bearing IFI35-overexpressing CT26 cells compared to tumors bearing control cells (Fig. 5G–L), suggesting reduced exhaustion. Taken together, these data provide evidence that IFI35 promoted intratumoral CD8^+^ T cell proliferation and enhanced CD8^+^ T cell function.

### Tumor IFI35 improves the efficacy of immunotherapy

**Fig. 6:**
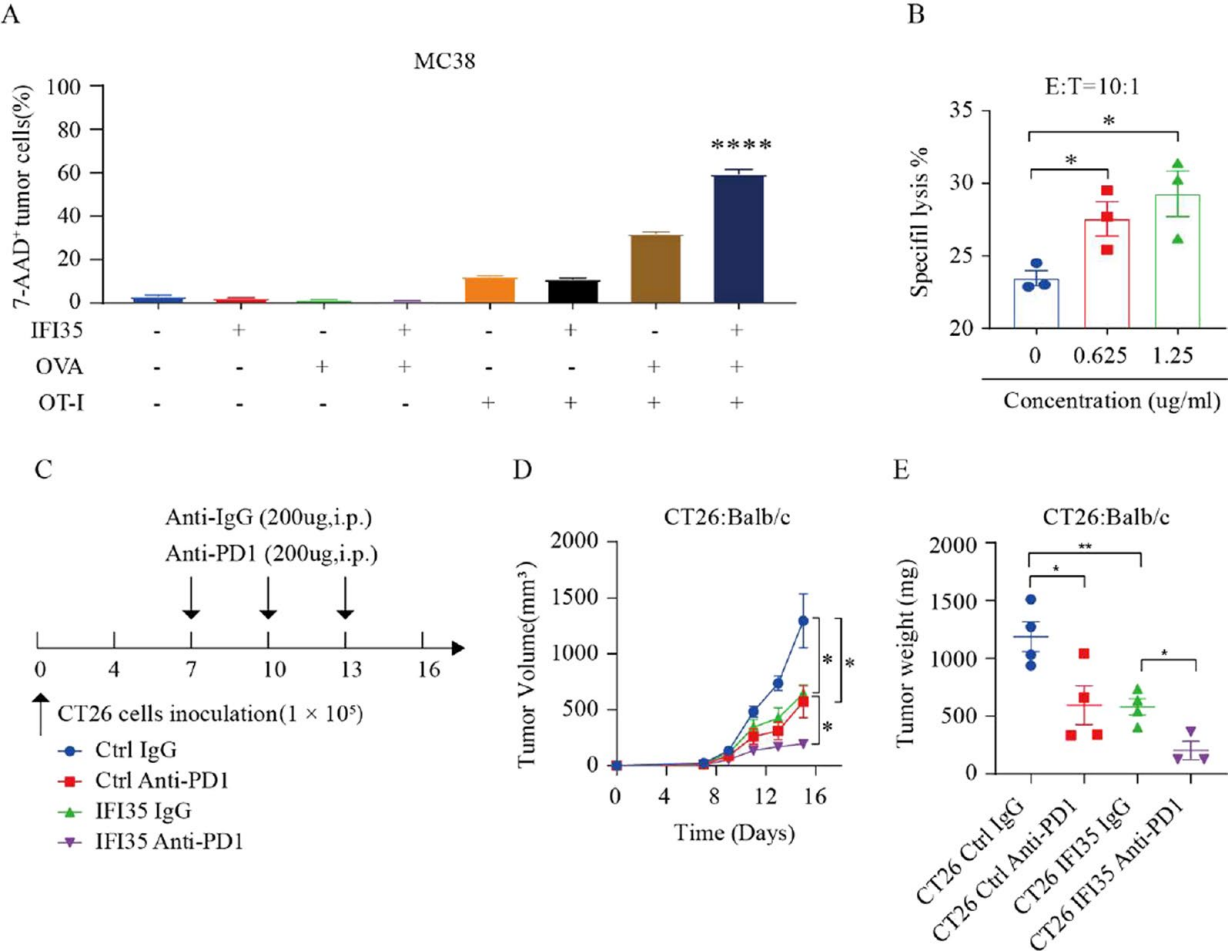
IFI35 enhanced immunotherapy efficacy of CRC. **A** Effect of tumor IFI35 on OT-I-mediated tumor killing. OVA expressing IFI35 MC38 cells were co-cultured with OT-I cells for 24 h. Tumor cell apoptosis was determined by flow cytometry analysis. Results are shown as the percentages of 7-AAD^+^ tumor cells. n = 3. Error bars represent the mean ± SEM. Two tailed t-tests, ****P < 0.0001. **B** Specific lysis of Lovo tumor cells after coculture with CAR T cells at a 10:1 effector/target (E/T) ratio for 4H, in the presence of 0, 0.625 and 1.25 µg/mL rhIFI35. n = 3. Error bars represent the mean ± SEM. Two tailed t-tests, *P < 0.05. **C**–**E** Overexpression of IFI35 improving the efficacy of anti-PD1 therapy. **C** The workflow of anti-PD1 therapy was shown. **D**, **E** CT26 cells were subcutaneously implanted into BALB/c mice and received anti-PD1 therapy. The tumor size and tumor weight are shown. n = 4 mice for CT26 Ctrl IgG groups, n = 4 mice for CT26 Ctrl Anti-PD1 groups, n = 4 mice for CT26 IFI35 IgG groups, n = 3 mice for CT26 IFI35 Anti-PD1 groups. Data are presented as means ± SEMs, *p < 0.05, **p < 0.01

The above results demonstrated that IFI35 slowed the progression of CRC in a CD8^+^ T cell-dependent manner. To access whether increasing the intratumoral proportion and enhancing function of CD8^+^ T cells in IFI35-overexpressing tumor resulted in improving efficacy of immunotherapy, we established multiple models combining with IFI35 overexpression, seeking to uncover that IFI35 may enhance the efficacy of immunotherapy. First, we conducted the OT-I transgenic mouse model and the CAR-T cell-mediated cytotoxicity assay to investigate the influence of IFI35 on antitumor immunity. CD8^+^ T cells were isolated from OT-I mice and activated with anti-CD3 (1 µg/mL), anti-CD28 (1 µg/mL), and IL-2 (10 ng/mL). Our results revealed that IFI35 significantly enhanced the cytotoxic effect of OT-I cell (Fig. 6A). Then, we evaluated CAR-T cell-mediated cytotoxicity through co-culture of target Lovo cancer cells and effector CAR-T cells which were preconditioned with rhIFI35 at different concentrations. We found that rhIFI35 significantly augmented the antitumor effect of CAR-T (Fig. 6B). Next, we evaluated if IFI35 overexpression could synergize immune checkpoint blockade therapy. Tumor growth delay experiments were carried out using a murine anti-PD1 immune checkpoint inhibitor in syngeneic mice inoculated with IFI35-overexpressing CT26 cells. Our study’s results demonstrate that the combination of anti-PD1 antibody treatment and IFI35 overexpression exhibited a synergistic effect in suppressing tumor growth (Fig. 6C–E). Overall, these observations suggest that IFI35 has outstanding potential in improving immune therapy against CRC.

### Tumor-secreted IFI35 activates CD8^+^ T cells through PI3K/AKT/mTOR pathway

**Fig. 7:**
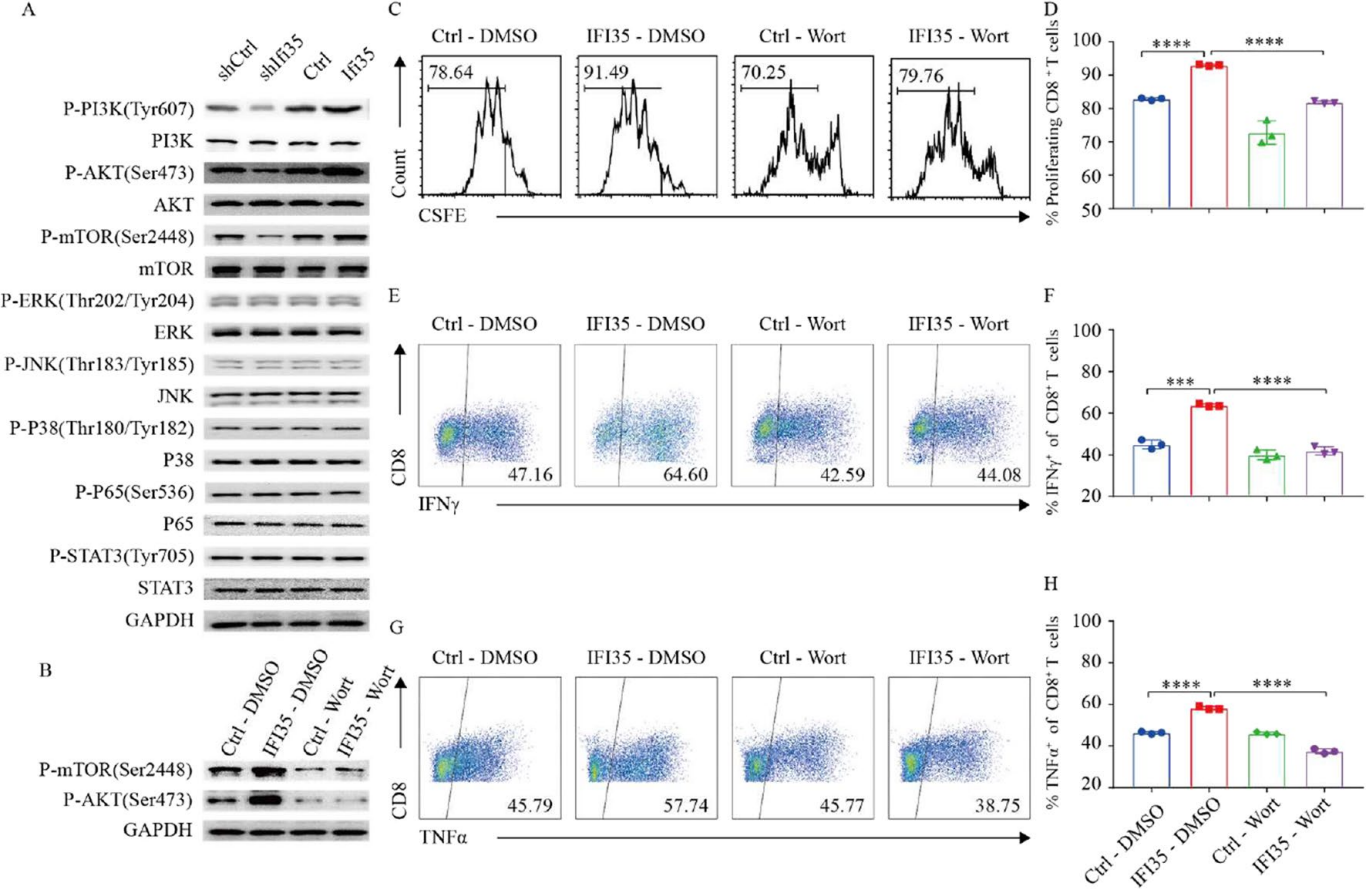
Tumor-secreted IFI35 protein activated CD8^+^ T cells thought PI3K/AKT/mTOR pathway. **A** Representative Western blots of p-mTOR, mTOR, p-AKT, AKT, p-ERK, ERK, p-JNK, JNK, p-P38, P38, p-P65, P65, p-STAT3, STAT3, and GAPDH in CD8^+^ T cells. CD8^+^ T cells stimulated with anti-CD3 (1 µg/mL), anti-CD28 (1 µg/mL), and IL-2 (10 ng/mL) were treated with supernatant from IFI35 knockdown or overexpression CT26 and MC38 cells for 2 days. **B,** Representative western blots of p-mTOR, p-AKT, and GAPDH in CD8^+^ T cells. The CD8^+^ T cells stimulated with anti-CD3 (1 µg/mL), anti-CD28 (1 µg/mL), and IL-2 (10 ng/mL) were pretreated with or without chemical inhibitors Wortmannin (20 nM) at 37 °C for 2 h. **C**, **D** The CFSE-labeled mouse CD8^+^ T cells were pretreated with or without wortmannin (20 nM) for 2 h. The cells were stimulated with anti-CD3 (1 µg/mL), anti-CD28 (1 µg/mL), and IL-2 (10 ng/mL) in supernatant from IFI35 and control vector expressing CT26 for 72 h. Cell divisions were then analyzed by flow cytometry. n = 3. Error bars represent the mean ± SEM. Two tailed t-tests, ***P < 0.001, ****P < 0.0001. **E**–**H** Flow cytometric analysis of IFNγ and TNFα of CD8^+^ T cells. CD8^+^ T cells were pretreated with or without wortmannin (20 nM) for 2 h and stimulated with anti-CD3 (1 µg/mL), anti-CD28 (1 µg/mL), and IL-2 (10 ng/mL) in supernatant from IFI35 and control vector expressing CT26 for 72 h. n = 3. Error bars represent the mean ± SEM. Two tailed t-tests, ***P < 0.001, ****P < 0.0001

It was established that several signaling pathways including MAPK, PI3K/AKT/mTOR, and JAK/STAT pathways play important roles on T cells [31–36]. To investigate whether IFI35-mediated CD8^+^ T cell activation is dependent on these signaling pathways, we examined the phosphorylation of ERK, JNK, and P38 that mediates many events downstream of the MAPK pathway, and of PI3K, AKT, mTOR in PI3K/AKT pathway, and P65 in NF-κB pathway, as well as of STAT3 in the JAK-STAT signaling pathway. Compared with the control, CD8^+^ T cells cultured with supernatant from IFI35-knockdown cells expressed lower levels of phosphorylation of PI3K (p-PI3K), AKT (p-AKT), and mTOR (p-mTOR), but not of P38 (p-P38), ERK (p-ERK), JNK (p-JNK), P65 (p-P65), or STAT3 (p-STAT3). Similarly, higher levels of phosphorylation of Tyr607 in PI3K, Ser473 in AKT, and Ser2448 in mTOR were detected in CD8^+^ T cells cultured with supernatant from IFI35-overexpressing cells compared with the control group (Fig. 7A). Our results indicated that IFI35 may enhance proliferation and cytotoxicity of CD8^+^ T cells through the PI3K/AKT/mTOR signaling pathway. To further test our hypothesis, we inhibited PI3K in CD8^+^ T cells using wortmannin, an inhibitor of phosphatidylinositol 3-kinase (PI3K). As shown in Fig. 7B, the inhibitor significantly inhibited the expression of p-AKT and p-mTOR. Moreover, wortmannin completely blocked the IFI35-mediated increase in p-AKT and p-mTOR (Fig. 7B). As expected, IFI35-induced CD8^+^ T cell proliferation (Fig. 7C, D) and cytokine secretion (Fig. 7E–H) were completely blocked by the inhibitor of PI3K, which suggests that PI3K/AKT/mTOR signaling pathway is required for IFI35-mediated activation of CD8^+^ T cells. Taken together, these results indicated that PI3K/AKT/mTOR pathway was indispensable for IFI35-mediated proliferation, and cytokine production of CD8^+^ T cells.

### IFNγ induces IFI35 via STAT1/IRF7

**Fig. 8:**
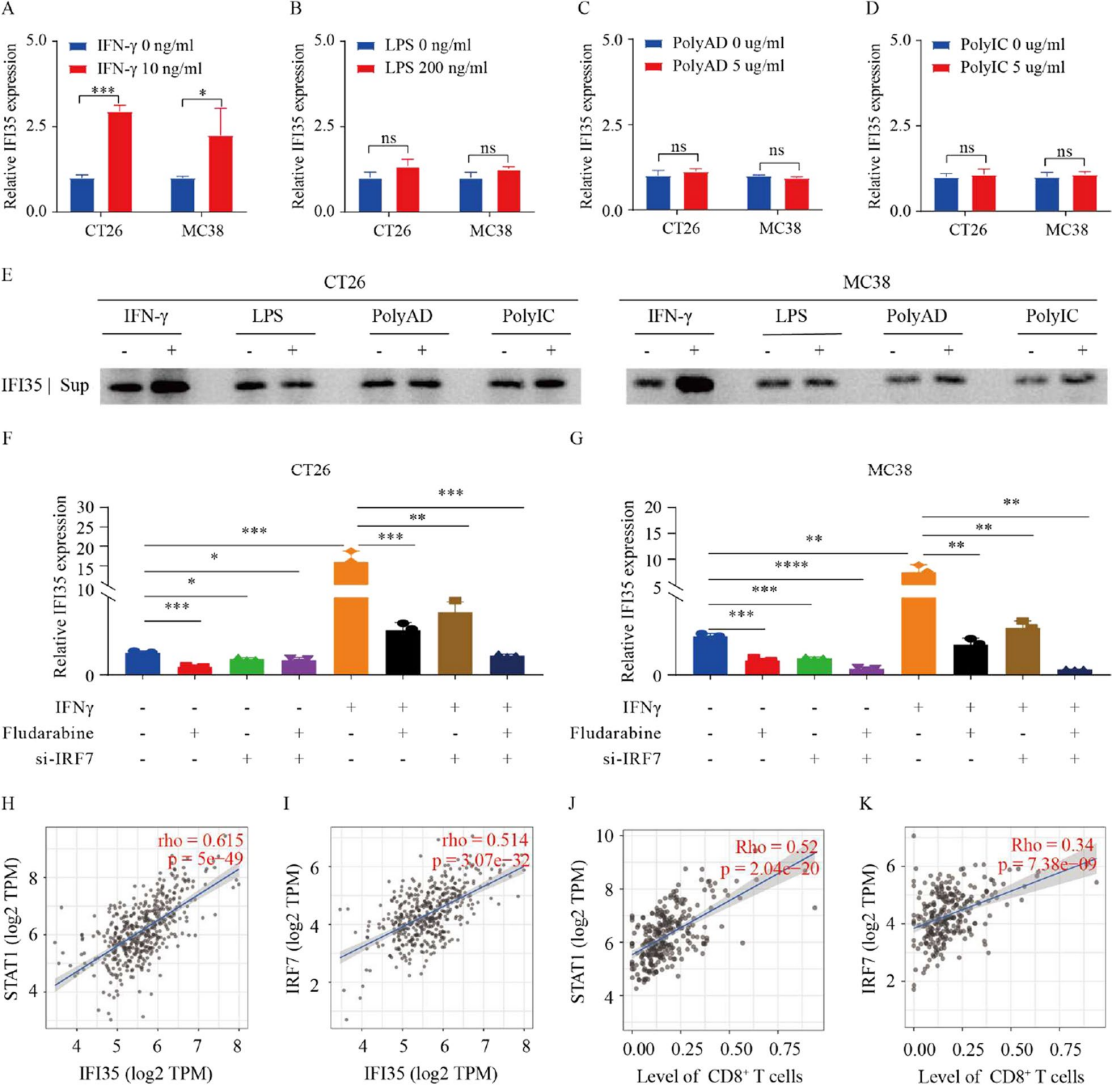
IFNγ induces IFI35 via STAT1/IRF7. **A**–**E** Treatment of murine colon cancer cells with IFNγ induced the expression of IFI35. **A**–**D** RNA was extracted from murine colon cancer cells after IFNγ (10 ng/mL), LPS (200 ng/mL), Poly AD (5 µg/mL), and poly IC (5 µg/mL) treatment for 16 h. n = 3. Error bars represent the mean ± SEM. Two tailed t-tests, *P < 0.05, ***P < 0.001. **E** Cell supernatant was subjected to western blot analysis for IFI35 after treatment with IFNγ (10 ng/mL), LPS (200 ng/mL), Poly AD (5 µg/mL), and poly IC (5 µg/mL) for 48 h. **F**, **G** Expression of IFI35 mRNA by RT-qPCR in CT26 and MC38 CRC cells transfected with control siRNA or one different siRNA sequence targeting IRF7 (si-IRF7) and then untreated or treated with IFNγ alone or in combination with STAT1 inhibitor Fludarabine. n = 3. Error bars represent the mean ± SEM. Two tailed t-tests, *P < 0.05, **P < 0.01, ***P < 0.001, ****P < 0.0001. **H**–**K** Correlation between IFI35 and STAT1 (**H**) or IRF7 (**I**) in COAD. Correlation between STAT1 (**J**) or IRF7 (**K**) expression and CD8^+^ T-cell expression in COAD

To characterize potential signals that govern IFI35 expression in CT26 and MC38 cells, we used different types of immune stimuli which may modulate IFI35 expression, including LPS, polyAD, polyIC, and IFNγ. In murine colon cancer cells challenged with IFNγ, both qPCR and WB analysis revealed increased expression of IFI35. However, no other stimuli induced the expression of IFI35 (Fig. 8A–E). STAT1 is a transcription factor downstream of IFNγ stimulation [37]. To explore whether STAT1 mediated the IFNγ-induced expression of IFI35, MC38 and CT26 cells were treated with fludarabine, a STAT1 inhibitor. Upon fludarabine treatment, decreased STAT1 mRNA levels in both murine colon cancer cells were observed (Additional file 1: Fig. S4A, B), and more importantly, the stimulated expression of IFI35 by IFNγ was attenuated (Additional file 1: Fig. S4C, D). These findings uncovered that IFNγ stimulates the expression of IFI35 via STAT1 signaling in murine colon cancer cells.

A group of transcription factors, IFN regulatory factors (IRFs) are activated by IFNγ [38]. Therefore we also assessed the potential role of IRFs in IFNγ stimulated IFI35 expression. First, we examined the expression of IRFs and found that, RNA expression of IRF1 and 7 was increased in both IFNγ-stimulated murine colon cancer cells (Additional file 1: Fig. S4E, F). We then successfully transfected the tumor cells with IRF expression plasmids, as demonstrated by over-expression of the IRFs (Additional file 1: Fig. S4G, H). Importantly, the overexpression of IRF7 significantly increased the expression of IFI35 in both murine colon cancer cell lines (Additional file 1: Fig. S4I, J). Thus, IRF7 was a unique transcription factor that was stimulated by IFNγ and drove the expression of IFI35 in both murine colon cancer cell lines.

To further verify the sequential roles of STAT1/IRF7 signaling in the IFNγ-induced IFI35 expression, we examined the changes in the expressions of IFNγ/STAT1/IRF7/IFI35 signaling molecules in IFNγ-stimulated murine colon cancer cells after pretreatment with STAT1 inhibitor fludarabine, si-IRF7 or both. The efficiency of si-IRF7 was verified by qPCR (Additional file 1: Fig. S4K, L). Here, induction of IRF-7 was no longer observed in IFNγ-stimulated MC38 and CT26 cells treated with STAT1 inhibitor (Additional file 1: Fig. S4M, N), indicating that IRF7 plays a role downstream of STAT1. Similarly, in the absence of STAT1 and/or IRF7, the expression of IFI35 were significantly decreased in the IFNγ stimulated cells (Fig. 8F, G). Our results demonstrated that IFNγ induces IFI35 expression by STAT1/IRF-7 signaling in CRC.

To determine the relevance of our findings in human Colon adenocarcinoma, we assessed IFNγ/STAT1/IRF7/IFI35/CD8 expression with Timer database [39–41]. The analysis showed that STAT1 and IRF7 expression levels were highly correlated with that of IFI35, as well as the number of CD8^+^ T cells in patients with COAD (Fig. 8H–K). Together, these results emphasized the clinical significance of the IFNγ/STAT1/IRF7/IFI35 axis in CRC.

## Discussion

Our data offer valuable insights into the tumor secreted protein, IFI35, which enhances the proliferation and cytotoxicity of CD8^+^ T cells by activating the PI3K/AKT/mTOR signaling pathway and suppresses tumor growth in a CD8^+^ T cell-dependent manner.

Up to now, the physiological and pathological functions of IFI35 in the cancer-immune environment are largely unknown. IFI35 regulates the proinflammatory cytokines including type I IFN [15, 42]. Notably, IFI35 has dual roles in viral infections dependent on viral species. Additionally, IFI35 activates microglia through the TLR4 pathway and promotes the differentiation of CD4^+^ T cells via DC activation [43]. All these studies support a role of IFI35 in immune regulation. In line with these, our study demonstrated that IFI35 plays a role in the immune environment of colorectal cancer. Since cytotoxic T-lymphocytes (CTLs) are associated with better response to immunotherapy [44], we started with data mining on CRC transcriptome in the TCGA and our CRC proteome data to delineate the relationship between IFI35 expression and CD8^+^ T cells. We found that IFI35 is positively associated with the activation of CD8^+^ T cells and predicts a better prognosis in multiple kinds of cancers, thus we hypothesized that IFI35 is involved in regulating the anti-tumor activity of CD8^+^ T cells.

Initially, we explored the relationship between IFI35 expression levels and the outcomes of patients with CRC. We revealed that high expression levels of IFI35 predicted a better prognosis, which was the first time to demonstrate a prognostic value of IFI35 for solid tumors. With cutaneous T-cell lymphoma, IFI35 was shown to be significantly down-regulated in the malignant cell population [16], which is consistent with our finding that IFI35 may suppress tumor growth. Subsequently, we examined the anti-tumor effect of IFI35 in vivo. We found that immunocompetent mice inoculated with IFI35-overexpressing murine colon cells exhibited stronger anti-tumor ability than the controls. IFI35 caused strong inhibition of tumor growth in immunocompetent mice, but not in nude mice, which suggested that the antitumor effect of IFI35 is immune-dependent. Then we evaluated the numbers and status of tumor-infiltrating immune cells by flow cytometry. Our findings revealed that IFI35 played a significant role in promoting the proliferation of CD8^+^ T cells and enhancing the function of cytotoxic CD8^+^ T cells. This was achieved by upregulating the expression of IFNγ and TNFα, while concurrently downregulating the expression of exhaustion markers. More importantly, we showed that IFI35 was secreted by tumor cells and promoted the proliferation and cytotoxicity of CD8^+^ T cells by activating the PI3K/AKT/mTOR signaling pathways. Our observation is different from the previous study, which concluded that IFI35 activated macrophages through the NF-κB pathway as a DAMP molecule to promote inflammation [15]. This difference indicates that the functions of IFI35 may be context-dependent.

The advent of immune checkpoint inhibitor (ICI) has yielded satisfactory results in cancer immunotherapy. Increasing the number of CD8^+^ T cells could enhance the efficacy of ICIs [8, 45]. Our study discovered that overexpressing IFI35 enhanced anti-PD-1 therapy in vivo. Other immunotherapies, such as chimeric antigen receptor T-cell (CAR-T) therapy, have also shown promising performance. Although CAR-T cell therapy has demonstrated significant efficacy with many haematological malignancies [46, 47], but not with solid tumors. Since tumor-secreted IFI35 could enhance cytotoxicity of CD8^+^ T cells, we wondered whether IFI35-stimulated CAR-T cells would do better in eliminating tumors. Our results showed that rhIFI35 significantly augmented the antitumor effect of CAR-T. In conclusion, our data unequivocally establish that IFI35 possesses substantial therapeutic potential for CRC.

We found that IFI35 was induced by IFNγ at both RNA and protein levels but not affected by LPS, poly IC, and poly A:D in murine colon cancer cells. It had been reported that stimulated macrophages could release IFI35 upon stimulation with lipopolysaccharide (LPS) or bacteria [15]. Moreover, Yu et al. found that macrophages and lung epithelial cells released IFI35 following influenza or SARS-CoV-2 virus infection [48]. What’s more, the TLR3/IFNβ/P-STAT1/RIG-I/CXCL10/CCL5 axis is negatively regulated by polyinosinic-polycytidylic acid (poly IC)-induced IFI35 in U373MG cells [49]. And various cells exhibit induced expression of IFI35 upon stimulation with IFNγ [10], which are consistent with our findings.

We next investigated the possible mechanism for IFNγ-induced IFI35 expression. It is known that the phosphorylation of STAT1 is induced by IFNγ binding to the IFNγ receptor complex. Then, phosphorylated STAT1 dimers induce the expression of interferon-stimulated genes (ISGs) and transcription of IFN regulatory factors (IRFs) in the nucleus [50]. Since IFI35 is an ISG and is induced by IFNγ, we postulated that IFNγ-activated STAT1 acts as an activator of the transcription of IRFs, which, in turn, induces the expression of IFI35. Our data have demonstrated that IFNγ enhanced IRF3/7 expression and upregulation of IRF7 increases the expression of IFI35 in both murine colon cancer cells. Consistent with these, expression of IFI35 was significantly decreased by IRF7 knockdown, and by inhibition of STAT1 with fludarabine. Importantly, we demonstrated that STAT1 activity is required for IRF7 expression in these cells. Further, we demonstrated positive correlations between the expression level of IFI35 and those of IFNG, STAT1, and IRF7. Importantly, patients with high expression levels of STAT1, IRF7, and IFI35 exhibited significantly more CD8^+^ T cell number. These results showed that IFNγ-STAT1-IRF7 signaling induced the expression of IFI35, which led to enhanced CD8^+^ T cells expression in murine colon cancer cells. This implies that IFI35 may play an essential role in regulating the colorectal cancer immune microenvironment in a CD8^+^ T cell dependent manner.

In summary, the tumor-secreted protein IFI35 plays crucial roles in the proliferation and cytotoxic activity of CD8^+^ T lymphocytes. IFI35 shows promising potential as a biomarker for predicting prognosis and guiding immunotherapy in CRC.

## Conclusion

In conclusion, our study has revealed that IFI35 not only has the potential to serve as a novel biomarker that enhances the proliferation and function of CD8^+^ T cells, but also represents a promising therapeutic target for colorectal cancer.

## Supplementary Information

**Additional file 1: Figure S1.** Additional data on the association of tumor IFI35 with CD8^+^ T cells expression and patient outcome. **A**, **B** Scatterplots of correlation between IFI35 and activated CD8^+^ T cells expression in CRC and solid cancersin published database. **C**–**H** Positive correlation of IFI35 mRNA levels with CD8^+^ T cells expression in human Adrenocortical carcinoma, Bladder Urothelial Carcinoma, Cervical squamous cell carcinoma and endocervical adenocarcinoma, Esophageal carcinoma, Head and Neck squamous cell carcinoma, Kidney Chromophobe, Liver hepatocellular carcinoma, Stomach adenocarcinoma. Data from the Timer database. **I**–**K** Higher levels of IFI35 expression correlated to better survival in 3 cancer patient cohorts including hepatocellular carcinoma, head and neck squamous cell carcinoma, and stomach adenocarcinoma. P values calculated by log-rank test. Data from public proteomics datasets. **Figure S2.** Gating strategy for immune cells population and additional data on intratumoral number of immune cells. **A** Live cells were selected by the live/dead dye. CD45^+^NKp64^+^ cells were defined as NK cells. CD45^+^CD19^+^ cells were defined as B cells. CD45^+^γδ T^+^ cells were defined as γδ T cells. CD8^+^ and CD4^+^ T lymphocytes were from the CD45^+^CD8^+^ and CD45^+^CD4^+^ subpopulation respectively. Tregs were subdivided from CD4^+^ T lymphocytes and were defined as CD45^+^ CD4^+^Foxp3^+^ population. DC were defined as the CD45^+^CD11b^+^CD11c^+^ subset. MDSC were defined as the Ly6G^+^Ly6C^low^ subpopulation of the CD45^+^CD11b^+^ subset. Total macrophages were defined as the F480^+^ subpopulation of the CD45^+^CD11b^+^ subset. **B**–**F** Effect of IFI35 overexpression on CT26 tumor-infiltrating various immune cells. IFI35 transfected CT26 cells were inoculated into BALB/c mice. The numbers of tumor-infiltrating γδ T cells, B cells, DC cells, NK cells, and MΦ cellswere analyzed by flow cytometry. Cell numbers are given for 1000,000 total cells from each tumor. Values are represented as mean ± SEM. P values were determined by two tailed t-tests. **P < 0.01, ns: not significant. **G**–**K** Effect of IFI35 overexpression on MC38 tumor-infiltrating various immune cells. IFI35 transfected MC38 cells were inoculated into C57/BL6 mice. The numbers of tumor-infiltrating γδ T cells, B cells, DC cells, NK cells, and MΦ cellswere analyzed by flow cytometry. Cell numbers are given for 1000,000 total cells from each tumor. Values are represented as mean ± SEM. P values were determined by two tailed t-tests. **P < 0.01, ns: not significant. **Figure S3.** The impact of IFI35 on the function of TAMs and Treg cells. **A** Gating strategy for detection of TAMs and exhaustion markers on the Treg cells by flow cytometry. Live cells were selected by the live/dead dye. CD45^+^CD11b^+^F480^+^CD86^+^ cells were defined as M1 macrophages. CD45^+^CD11b^+^F480^+^CD206^+^ cells were defined as M2 macrophages. **B**, **C** Proportions of M1and M2macrophages in the TME of BALB/c mice injected subcutaneously with vector control and IFI35-overexpressing CT26 cell line. Values are represented as mean ± SEM. P values were determined by two tailed t-tests. ns: not significant. **D**–**F** Proportions of exhaustion markers on the Treg cells in the TME of BALB/c mice injected subcutaneously with vector control and IFI35-overexpressing CT26 cell line. quantification of LAG3, CD39and CTLA4expression among Treg cells. Values are represented as mean ± SEM. P values were determined by two tailed t-tests. ns: not significant. **Figure S4.** Impact of pharmacological inhibition of STAT1 and genetic inhibition of IRF7 on the expression of STAT1, IFI35, and IRF7 in murine colon cancer cells. **A**–**D** the expression of STAT1 and IFI35 mRNA by RT-qPCR in MC38and CT26colorectal cancer cells treated with or without STAT1 inhibitor or/and IFNγ. n = 3. Error bars represent the mean ± SEM. Two tailed t-tests, *P < 0.05, **P < 0.01, ***P < 0.001, ****P < 0.0001. **E**, **F** the expression of IRFs mRNA was measured after CT26and MC38were treated with 10 ng/mL IFNγ for up to 16 h. n = 3. Error bars represent the mean ± SEM. Two tailed t-tests, *P < 0.05, **P < 0.01, ***P < 0.001, ****P < 0.0001. **G**–**J** Expression of IRF1, IRF7 and IFI35 mRNA by RT-qPCR in the CT26 and MC38 CRC cells transfected with IRF1 or IRF7 plasmid. n = 3. Error bars represent the mean ± SEM. Two tailed t-tests, *P < 0.05, **P < 0.01, ****P < 0.0001. **K**, **L** the expression of IRF7 mRNA by RT-qPCR in controlor Irf7-targetedMC38and CT26colorectal cancer cells. n = 3. Error bars represent the mean ± SEM. Two tailed t-tests, *P < 0.05, ***P < 0.001. **M**, **N** the expression of IRF7 mRNA by RT-qPCR in MC38and CT26colorectal cancer cells treated with or without STAT1 inhibitor or/and IFNγ. n = 3. Error bars represent the mean ± SEM. Two tailed t-tests, **P < 0.01, ****P < 0.0001.

**Additional file 2: Table S1.** PCR primers.

## Data Availability

The datasets used and/or analyzed during the current study are available from the corresponding author upon reasonable request.

## Abbreviations

CTLs: Cytotoxic T lymphocytes
IFI35: Interferon-induced protein-35 kDa
LPS: Lipopolysaccharide
TCGA: The Cancer Genome Atlas
CYT: Cytolytic activity
TAMs: Tumor-associated macrophages
LAG‐3: Lymphocyte activation gene‐3
CTLA‐4: Cytotoxic T-lymphocyte-associated protein 4
ELISA: Immunosorbent assay
ICI: Immune checkpoint inhibitor
CAR-T: Chimeric antigen receptor T-cell
ISGs: Interferon-stimulated genes
IRFs: IFN regulatory factors
