# Supplementary figures and images for: Tumor-secreted IFI35 promotes proliferation and cytotoxic activity of CD8+ T cells through PI3K/AKT/mTOR signaling pathway in colorectal cancer
Source: J Biomed Sci. 2023 Jun 28;30:47. doi: 10.1186/s12929-023-00930-6 (PMC10303345; doi:10.1186/s12929-023-00930-6)

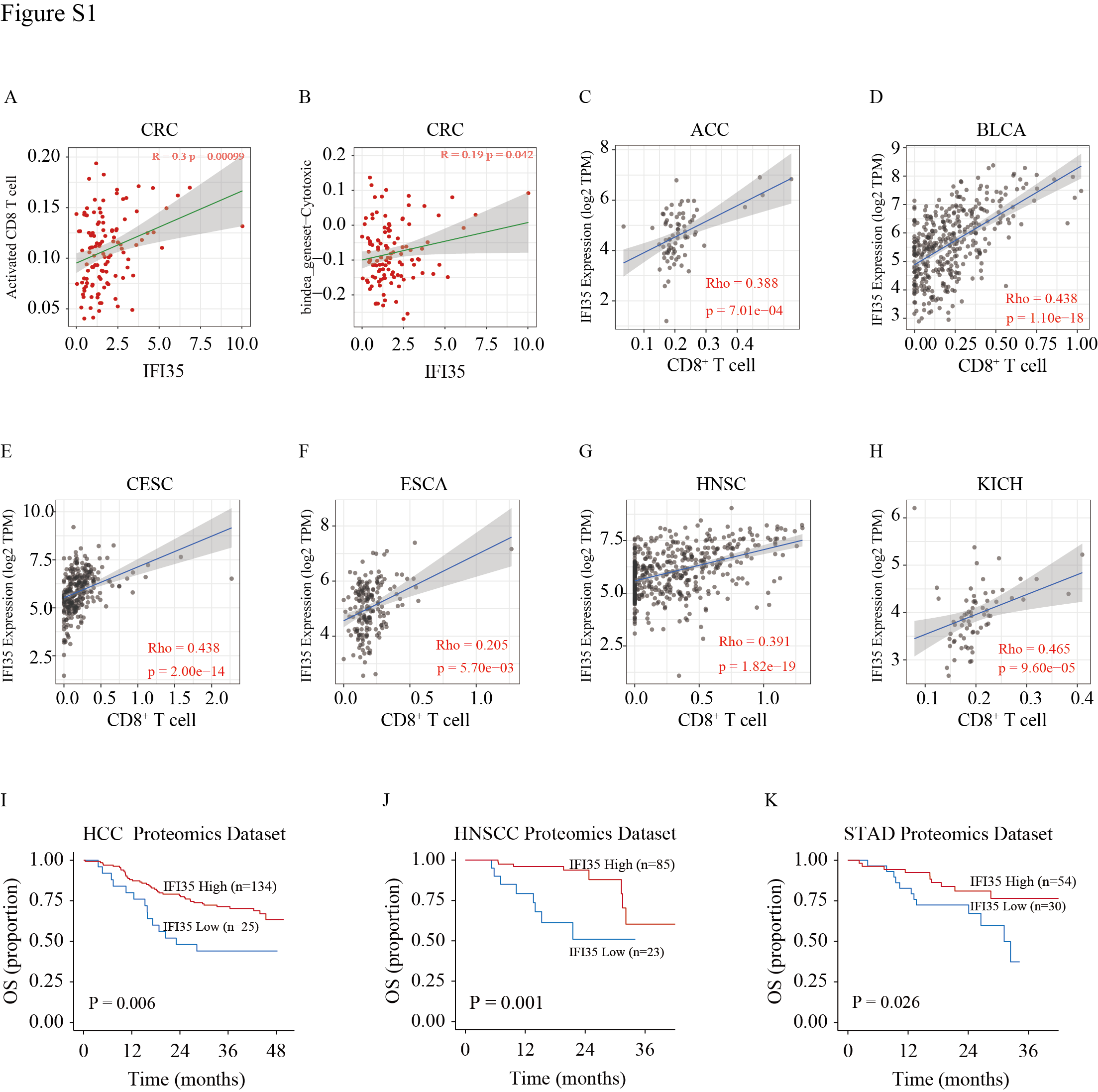

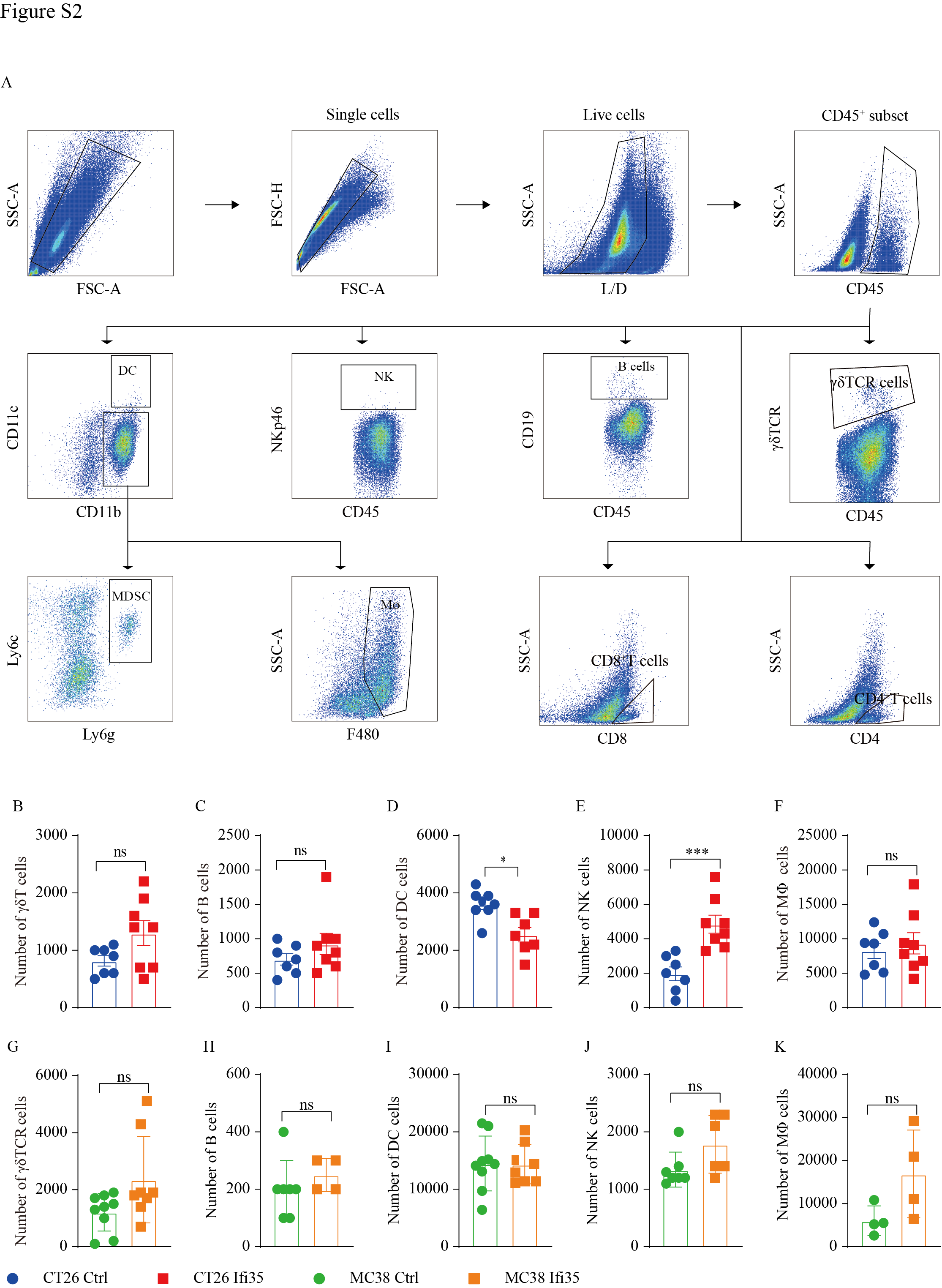

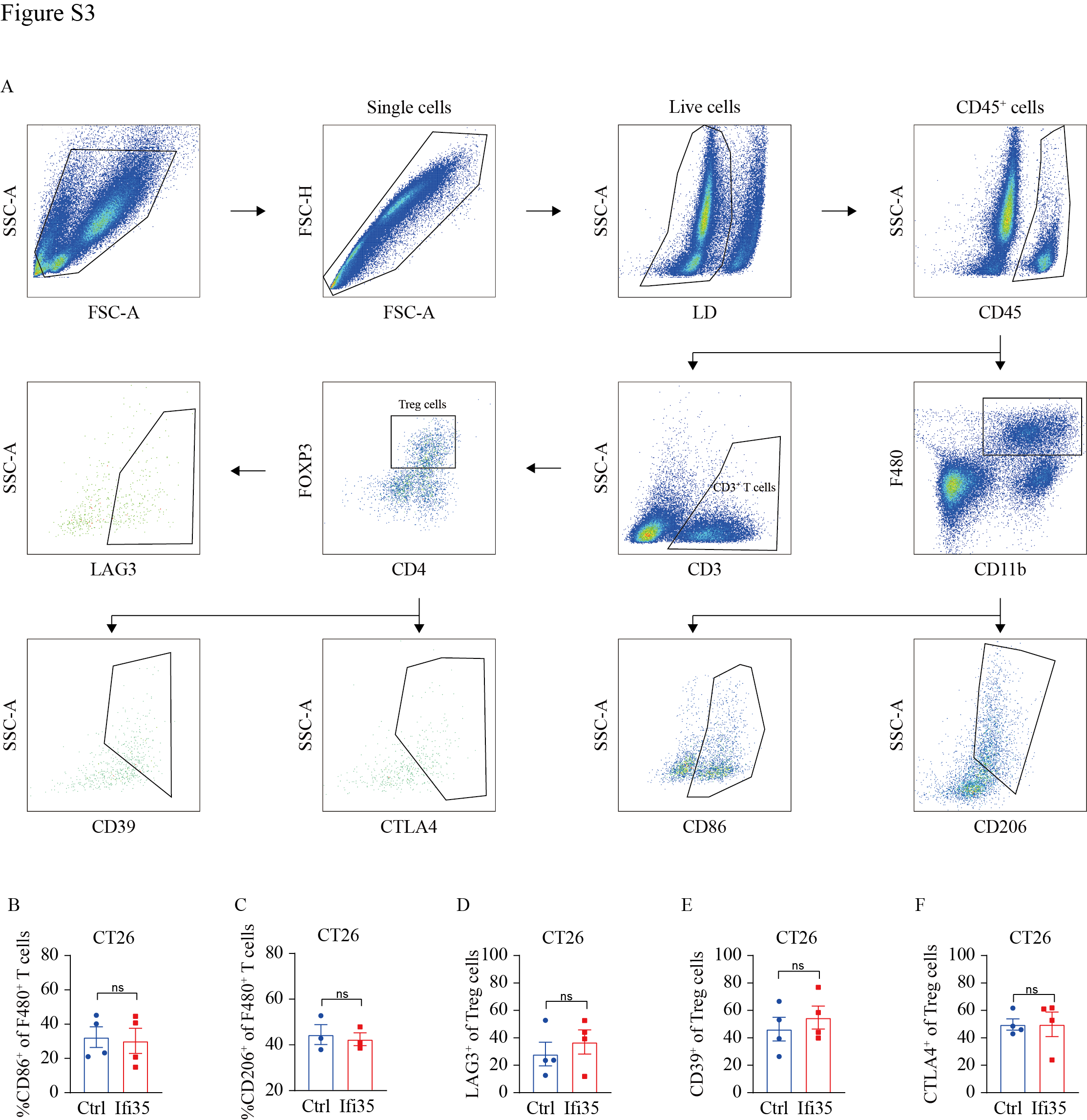

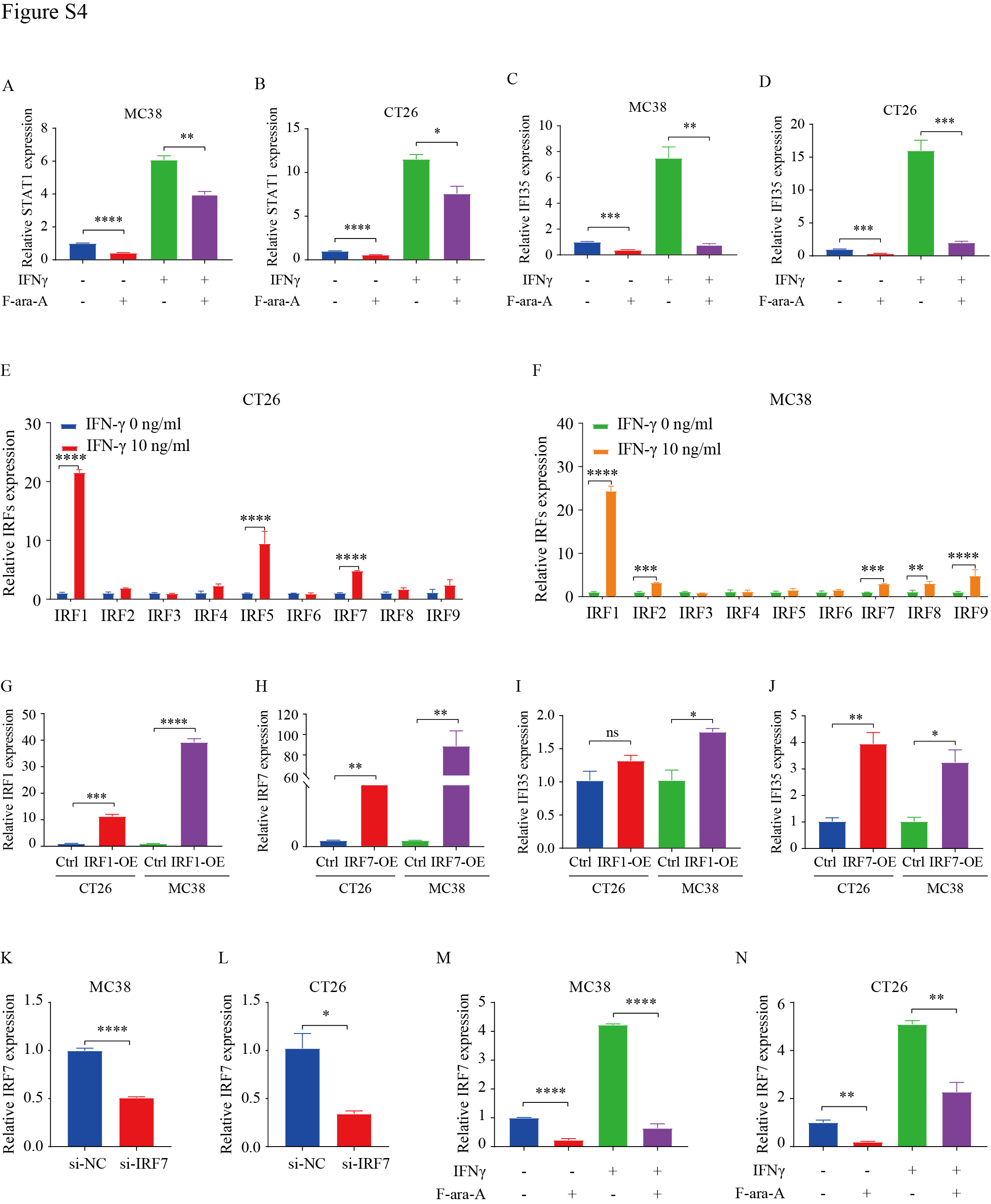

Supplement: Supplementary file 1 — Additional file 1: Figure S1. Additional data on the association of tumor IFI35 with CD8+ T cells expression and patient outcome. A, B Scatterplots of correlation between IFI35 and activated CD8+ T cells expression in CRC and solid cancersin published database. C–H Positive correlation of IFI35 mRNA levels with CD8+ T cells expression in human Adrenocortical carcinoma, Bladder Urothelial Carcinoma, Cervical squamous cell carcinoma and endocervical adenocarcinoma, Esophageal carcinoma, Head and Neck squamous cell carcinoma, Kidney Chromophobe, Liver hepatocellular carcinoma, Stomach adenocarcinoma. Data from the Timer database. I–K Higher levels of IFI35 expression correlated to better survival in 3 cancer patient cohorts including hepatocellular carcinoma, head and neck squamous cell carcinoma, and stomach adenocarcinoma. P values calculated by log-rank test. Data from public proteomics datasets. Figure S2. Gating strategy for immune cells population and additional data on intratumoral number of immune cells. A Live cells were selected by the live/dead dye. CD45+NKp64+ cells were defined as NK cells. CD45+CD19+ cells were defined as B cells. CD45+γδ T+ cells were defined as γδ T cells. CD8+ and CD4+ T lymphocytes were from the CD45+CD8+ and CD45+CD4+ subpopulation respectively. Tregs were subdivided from CD4+ T lymphocytes and were defined as CD45+ CD4+Foxp3+ population. DC were defined as the CD45+CD11b+CD11c+ subset. MDSC were defined as the Ly6G+Ly6Clow subpopulation of the CD45+CD11b+ subset. Total macrophages were defined as the F480+ subpopulation of the CD45+CD11b+ subset. B–F Effect of IFI35 overexpression on CT26 tumor-infiltrating various immune cells. IFI35 transfected CT26 cells were inoculated into BALB/c mice. The numbers of tumor-infiltrating γδ T cells, B cells, DC cells, NK cells, and MΦ cellswere analyzed by flow cytometry. Cell numbers are given for 1000,000 total cells from each tumor. Values are represented as mean ± SEM. P values were determine [file 12929_2023_930_MOESM1_ESM.docx]
