# Supplementary material for: Tumor-secreted IFI35 promotes proliferation and cytotoxic activity of CD8+ T cells through PI3K/AKT/mTOR signaling pathway in colorectal cancer
Source: J Biomed Sci. 2023 Jun 28;30:47. doi: 10.1186/s12929-023-00930-6 (PMC10303345; doi:10.1186/s12929-023-00930-6)
Supplement: Supplementary file 2 — Additional file 2: Table S1. PCR primers. [file 12929_2023_930_MOESM2_ESM.docx]

Table S1. PCR primers

| **Target Gene** | **Forward primer** | **Reverse primer** |
| --- | --- | --- |
| Mouse Ifi35 | CAAGCCAGGCTCAAGATGAGG | TGGCCTTGGAATACCAGAGGA |
| Mouse β-Actin | CATTGCTGACAGGATGCAGAAGG | TGCTGGAAGGTGGACAGTGAGG |
| Mouse Irf1 | ATGCCAATCACTCGAATGCG | CCTGCTTTGTATCGGCCTGT |
| Mouse Irf2 | GTGGAACGGATGCGAATGC | CAGCCACTTTAGCCCTGGTAT |
| Mouse Irf3 | GAGAGCCGAACGAGGTTCAG | CTTCCAGGTTGACACGTCCG |
| Mouse Irf4 | CCGACAGTGGTTGATCGACC | CCTCACGATTGTAGTCCTGCTT |
| Mouse Irf5 | GGTCAACGGGGAAAAGAAACT | CATCCACCCCTTCAGTGTACT |
| Mouse Irf6 | CTCTCCCCATGACTGACTTGG | CAGGTCCCCATAGAAGAGCC |
| Mouse Irf7 | GAGACTGGCTATTGGGGGAG | GACCGAAATGCTTCCAGGG |
| Mouse Irf8 | CGGGGCTGATCTGGGAAAAT | CACAGCGTAACCTCGTCTTC |
| Mouse Irf9 | GCCGAGTGGTGGGTAAGAC | GCAAAGGCGCTGAACAAAGAG |
| Mouse Stat1 | TCACAGTGGTTCGAGCTTCAG | GCAAACGAGACATCATAGGCA |
